# Pooled analyses of Clostridioides difficile vaccine trials identify baseline predictors for vaccine response

**DOI:** 10.1038/s41598-026-42375-5

**Published:** 2026-03-12

**Authors:** Igor Stojkov, Linda Marchioro, Isabelle Bekeredjian-Ding, Benjamin Hofner

**Affiliations:** 1 Section Data Science and Methods, Paul-Ehrlich-Institut, Langen, Germany; 2 Division of Microbiology, Paul-Ehrlich-Institut, Langen, Germany; 3 Institute for Medical Microbiology and Hospital Hygiene, Philipps-University of Marburg, Marburg, Germany; 4 Department of Medical Informatics, Biometry and Epidemiology, Friedrich-Alexander-Universität Erlangen-Nürnberg, Erlangen, Germany

**Keywords:** Diseases, Gastroenterology, Health care, Immunology, Medical research, Microbiology

## Abstract

*Clostridioides difficile* (*C. difficile*) is a major cause of severe diarrhea and colitis usually affecting individuals with dysbiosis. Currently, there is no licensed vaccine nor prophylaxis for *C. difficile* infection (CDI), while treatment remains challenging. To support immunogenicity assessment and vaccine development programs, we analyzed data from two Sanofi vaccine trials (Phase II and III) with 1,096 participants. We evaluated baseline predictors of 16 seroresponse outcomes, measured 30 days after the third vaccine dose, using five statistical models. Relevant predictors of positive seroresponse after vaccination for both Toxin A and B included lower comorbidity index, age < 65 years, and future CDI risk exposure (i.e., impending hospitalization/nursing facility admission were more relevant than past-exposure history). Higher baseline antibody levels, North American study region, and female sex were mainly related with Toxin B seroresponse. These findings highlight the need for further research to optimize vaccine trial design and personalized vaccination approaches.

**Supplementary Information:**

The online version contains supplementary material available at 10.1038/s41598-026-42375-5.

## Introduction

*Clostridioides difficile* (*C. difficile*), formerly known as *Clostridium difficile* or *Bacillus difficile*, is a Gram-positive, anaerobic, rod-shaped bacterium, capable of forming resistant spores and commonly found in the environment, and intestinal tracts of animals and humans^1,2^. In the human population, asymptomatic colonization rates for toxigenic or non-toxigenic strains are high and vary across age groups with estimated 41% in infants, 12% in children (5–18 years), and around 18% in adults^3,4^. Toxigenic *C. difficile* colonization ranges from 14% in infants, 6% in children, and around 8% in adults, without clinical symptoms^3,5^. These percentages are often higher for those exposed in healthcare or long-term nursing facilities, and thus contribute to an overall asymptomatic transmission potential^6,7^.

*C. difficile* transmission occurs primarily through the fecal-oral route. Disruptions of natural immunity and colonic microbiota are prime contributors in the multifactorial setting that can lead to further *C. difficile* propagation in the large intestine^2,8^. This can result in a *C. difficile* infection (CDI), mainly characterized by the production and activity of exotoxin A (TcdA) and/or exotoxin B (TcdB) that damage the intestinal epithelial cells and trigger inflammatory responses. The resulting clinical manifestations range from mild/moderate diarrhea to severe colitis, and may lead to potential life-threatening complications^9,10^.

*C. difficile* is widely recognized as the primary cause of antibiotic-related diarrhea in healthcare facilities^11,12^. However, it is not limited to this setting, as it is increasingly associated with cases of colitis in the wider outpatient community. In the recent 2022 surveillance report from the United States Centers for Disease Control and Prevention, there was an estimated incidence of 116.1 cases of CDI per 100,000 people per year, with 62.1 cases attributed to community-acquired incidence^13^. Significant increases in CDI burden were reported in Europe, particularly for the period prior to the COVID-19 pandemic^14,15^, as well as in Australia^16^ and Asia^16,17^. And while *C. difficile* is not part of the 2024 WHO Bacterial Priority Pathogens List^18^, it is widely acknowledged as a significant global public health concern with substantial health and economic burden^19,20^.

CDI treatment can be challenging in terms of finding an effective and safe therapy and preventing recurrence^21^. Antibiotic treatment using metronidazole, vancomycin, and fidaxomicin are primary choices for CDI treatment, but have little effect on dysbiosis and cannot reliably prevent recurrence. Bezlotoxumab, a human monoclonal antibody targeting TcdB, and fecal microbiota transplants represent treatment options indicated for recurrent CDI. Furthermore, antibiotic resistance in CDI patients is an increasing concern, as emerging strains (e.g., BI/NAP1/027) display reduced susceptibility to current treatments, requiring more complex treatment strategies and associated with worse prognosis^22,23^. Moreover, as passive immunity decreases after administration of a therapeutic antibody, a more effective and long term strategy could be to prioritize active immunity^24^.

Significant efforts have been made towards development of a safe and effective vaccine capable of inducing high levels of neutralizing antibodies against TcdA and TcdB that could prevent CDI, achieve valuable health benefits and reduce health expenditures^24–26^. Studies in animals and humans have established that fecal and serum immunglobulin A (IgA) and IgG directed against the *C. difficile* toxins are protective, and justify their use as an immunological correlate of protection for vaccine development^27–29^. However, despite substantial efforts by multiple companies, there is no approved *C. difficile* vaccine available. Sanofi Pasteur’s vaccine development program for *C. difficile* was based on formalin-inactivated TcdA and TcdB and underwent testing in three Phase I and two Phase II trials. Despite positive outcomes in the earlier phases, the Phase III trial from 2013 was terminated due to failure to achieve the primary objective of preventing CDI cases^30^. Similarly, Pfizer’s *C. difficile* vaccine program with modified TcdA and TcdB, comprised two trials for Phase I and Phase II respectively, and a Phase III trial initiated in 2017 that did not meet its predefined primary endpoint of preventing CDI^31^. Valneva’s VLA84, based on a recombinant chimeric protein containing segments from the C-terminal ends of TcdA and TcdB, also faced challenges in its development. Following a Phase I and a Phase II trial in 2015, the program did not yet proceed to Phase III^32,33^. Moreover, GlaxoSmithKline is developing vaccine based on the TcdA fragment F2, which recently concluded a Phase I trial with promising safety and immunogenicity profile^34^. While toxin-neutralizing antibodies are primarily indicated for prophylaxis of CDI or relapse, they do not inhibit the pathogens’ ability to colonize the intestine. Thus, additional strategies currently applied in early development focus on vaccines targeting surface antigens required for colonization by vegetative cells and for prevention of spore formation^24,35^.

It is interesting to note that in the early phases, all vaccine candidates achieved positive results for immunogenicity (i.e. immune response to vaccine antigens). These results could, however, not be translated to clinical outcomes in later stage trials. Given the widely accepted correlation of vaccine efficacy and immunogenicity, understanding the factors that influence a vaccines’ ability to stimulate an immune response, are highly important for development and evaluation of candidate vaccines^36^. These factors may include differences based on age, sex, genetics, immunological parameters, behavioral factors, nutrition and overall health status, vaccine-specific characteristics etc.^37,38^. To our knowledge, to date, no published study has extensively analyzed the impact of intrinsic host factors on immune parameters in vaccine *C. difficile* trials. Therefore, our aim was to explore the association between different baseline characteristics and seropositive outcomes, to ultimately enhance patients’ benefit, utilizing the enriched pooled data from two randomized controlled trials of Sanofi’s *C. difficile* vaccine development program.

## Methods

Access to the data was granted by Sanofi Pasteur, and data were retrieved and analyzed via the Vivli platform^39^. We created a pooled dataset from two related vaccine trials (ClinicalTrials.gov identifier NCT01230957 - Phase II trial; NCT01887912 - Phase III trial stopped for futility), which tested formalin-inactivated TcdA and TcdB vaccine antigens against *C. difficile*. All participants who met the protocol-defined inclusion criteria for immunogenicity analyses in each trial were included in the pooled analysis. Adherence to protocols required participants to fulfill all eligibility criteria, receive the correct vaccine formulation and all three scheduled doses, and provide valid serology samples within the specified time windows. Participants were excluded from the per-protocol immunogenicity analyses if they failed to meet the study eligibility criteria (e.g., reported current or prior CDI episode, previous vaccination against *C.* *difficile* etc.), did not complete the vaccination schedule as assigned, received medications or vaccines that could affect the immune response, were given a vaccine not prepared or administered according to the protocol, or had missing or invalid serology result. Further details on the criteria for the per-protocol immunogenicity set are provided in the respective publications of the two trials^30,40^.

We used descriptive statistics to summarize the demographic and clinical characteristics of the participants, including frequencies and percentages for categorical variables and median with interquartile range for continuous variables. The following baseline characteristics of the pooled data set were examined as potential predictors of the post-vaccination immunogenicity (i.e., seroresponse): age, sex, comorbidity index, baseline immunogenicity, CDI risk exposure, and study region. The vaccine trial arms were naturally selected as an important adjustment variable in all models due to the expected strong effect.

The predictors were organized and combined in the pooled data based on the common variable characteristics within the two trials (e.g., merging variables using the same/similar age category, study region, vaccine trial arm). The “Study region” variable was used as provided with minor modifications (Supplementary Information), and the “Rest of the world” subcategory, as stated in the related Phase III trial publication^30^, included participants from Latin America and the Asia-Pacific region. Additionally, the baseline IgG values were dichotomized based on the median distribution values per trial, whereas the comorbidity index was newly derived using the Charlson comorbidity index guidance^41,42^ and the available text-based diagnoses from participants (Supplementary Tables 16–17). Seroresponse was measured using enzyme-linked immunosorbent assay (ELISA) and toxin neutralization assay (TNA), for both TcdA and TcdB. We focused on the seroresponse measurements taken 30 days after the final vaccination, reflecting the peak antibody response for each schedule: day 60 for the 0, 7, 30 day regimen, and day 210 for the 0, 7, 180 and 0, 30, 180 day regimens. These data were then combined to create a single pooled dataset. Since there is currently no established benchmark for determining a protective immune response in *C. difficile*, we used multiple seroresponse outcomes for the analyses. The cut-off points were defined as minimum two-fold, four-fold, median, and 75th percentile fold-increase from baseline. While the two-fold and four-fold increase criteria are commonly used, the median and 75th percentile increase cut-off points, which were derived in a data-driven manner from the fold-increase distribution within each trial and vaccine trial arm, were employed to identify participants with the strongest antibody responses and to provide a broader assessment of immunogenicity. As we separately analyzed ELISA and TNA measurements for each of the TcdA and TcdB, a total of 16 outcomes were investigated.

To assess the potential effect of the predictors, we used five different statistical models: full-model logistic regression, stepwise logistic regression, mixed-effect models^43^, classification and regression trees (CART)^44^, and boosting algorithms^45,46^. Associations were deemed relevant if they had a p-value < 0.05 in the first three model classes, or if the variable importance was at least 0.001 in the other two model classes. Variable importance was measured by the decrease in impurity in the CART models and by the in-bag risk reduction in the boosting models. To ensure robustness and comparability of our findings, we conducted an additional analysis of the variable importance using a model-agnostic approach. This involved measuring the impact of permuting variables on the model performance using the same loss function (i.e., one minus the area under the curve-AUC) across all models. If a variable was relevant for the prediction, permuting it should decrease the model prediction accuracy, demonstrating the significance of the variable in the model. Furthermore, we evaluated the discrimination ability of each model based on the AUC. To minimize bias in the assessment we split the data set into a training set, containing 75% of the pooled data, and a test set, containing the remaining 25% of data^47^. We fitted the models on the training data and evaluated them on the test data. All analyses were conducted using Stata software version 17 (StataCorp, College Station, TX) and R Statistical Software (version 4.2.2; R Foundation for Statistical Computing, Vienna, Austria). Further details on the data organization and statistical analyses are available in the Supplementary Data structure and analyses.

### Ethical aspects

The study was conducted in line with the Paul-Ehrlich-Institut’s guidelines for good scientific practice. The analyses were documented in a study plan, which was part of the written data access agreement between the Paul-Ehrlich-Institut and Sanofi Pasteur in order to obtain the anonymized data. Data were accessed through the Vivli platform, ensuring compliance with the General Data Protection Regulation. Since our study is based on secondary analyses using anonymized data, the Ethics Committee of the Faculty of Medicine, Goethe University Frankfurt am Main, waived the requirement for ethical approval and additional informed consent (Ref. 2025–2545). Written informed consent was obtained from each participant before enrollment in the original trials (NCT01230957 and NCT01887912), which were according to Sanofi Pasteur^30,40^ conducted in accordance with applicable local and national regulations, the International Conference on Harmonization Good Clinical Practice guidelines, and the Declaration of Helsinki.

## Results

### Analyzed (pooled) data

**Fig. 1:**
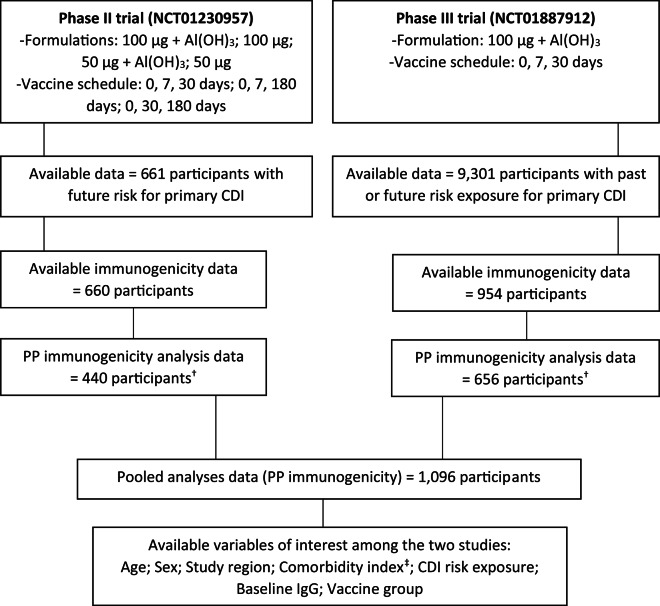
Structure of the pooled dataset. Note: *Al(OH)*_*3*_ - Aluminium hydroxide; *CDI* - Clostridioides difficile infection; *IgG* - Immunoglobulin G; *PP* - Per-protocol. ^†^Available PP immunogenicity data are used as defined in the respective PP immunogenicity datasets of each trial^30,40^. ^‡^The Charlson comorbidity index (modified), was newly derived based on the participants’ age and available text-based diagnoses.

The pooled dataset used for analysis consisted of 1,096 participants, including 440 from the Phase II trial and 656 from the Phase III trial, same as the immunogenicity datasets in the respective trial-related publications (Fig. 1). In the pooled dataset, participants were well balanced in terms of age and sex, with 46% over the age of 65 and 48% female (Table 1). The majority of the participants (68%) was from North America. Most participants (57%) received the 100 µg dose with adjuvant, whereas roughly one fourth (24%) received placebo, and only a minority (6–7%) was enrolled in each of the other three vaccine groups. In terms of the CDI risk exposure, around 63% were classified as future risk group with a low to medium comorbidity index (median 3, interquartile range 2–4). The baseline values for IgG against TcdA were relatively low and narrowly distributed, with quartiles being identical to the median. In contrast, baseline IgG levels for TcdB had wider distributions, mainly for ELISA (Supplementary Table 1). The median values from the seroresponse outcomes, which were also used for deriving the outcome variables, differed between the two trials with participants in the Phase III trial generally exhibiting lower IgG seroresponse levels compared to those in Phase II. For example, at the 60-day measurement, the median fold increases from baseline for Toxin A were 42 (ELISA) and 22 (TNA) in the Phase II trial, compared to 12 (ELISA) and 6 (TNA) in the Phase III trial. For Toxin B, Phase II results showed median fold increases of 55 (ELISA) and 6 (TNA), whereas Phase III results were lower, at 3 (ELISA) and 1 (TNA). This discrepancy is potentially attributable to the older age and medical history of participants in the Phase III trial, as previously discussed^30^. Additional details are presented in Supplementary Table 2.

**Table 1:** Summary characteristics of the pooled analyses dataset.

Participants’ characteristics	Participants = 1,096 *n* (%) / Median [IQR]
Age (> 65 years)	505 (46.1)
Sex (female)	525 (47.9)
Study region	
North America	745 (68.0)
Western Europe	80 (7.3)
Rest of the world*	271 (24.7)
CCI (continuous, score unit)	3 [2–4]
CDI risk (future risk)	690 (63.0)
Vaccine group	
100 µg + Al(OH)_3_	627 (57.2)
100 µg	73 (6.7)
50 µg + Al(OH)_3_	70 (6.4)
50 µg	68 (6.2)
Placebo	258 (23.5)
ELISA - Toxin A IgG	
Baseline IgG (above median)	164 (15.1)
Missing data	8 (0.7)
Seroresponse (Two-fold increase)	794 (73.1)
Seroresponse (Four-fold increase)	771 (71.0)
Seroresponse (Median fold increase)	571 (52.5)
Seroresponse (75th percentile fold increase)	286 (26.3)
Missing data	9 (0.8)
TNA - Toxin A IgG	
Baseline IgG (above median)	140 (12.9)
Missing data	7 (0.6)
Seroresponse (Two-fold increase)	745 (68.4)
Seroresponse (Four-fold increase)	702 (64.5)
Seroresponse (Median fold increase)	569 (52.3)
Seroresponse (75th percentile fold increase)	289 (26.5)
Missing data	7 (0.6)
ELISA - Toxin B IgG	
Baseline IgG (above median)	506 (46.4)
Missing data	5 (0.5)
Seroresponse (Two-fold increase)	748 (68.6)
Seroresponse (Four-fold increase)	684 (62.7)
Seroresponse (Median fold increase)	574 (52.6)
Seroresponse (75th percentile fold increase)	281 (25.8)
Missing data	5 (0.5)
TNA - Toxin B IgG	
Baseline IgG (above median)	240 (22.1)
Missing data	8 (0.7)
Seroresponse (Two-fold increase)	434 (39.9)
Seroresponse (Four-fold increase)	397 (36.5)
Seroresponse (Median fold increase)	441 (40.5)
Seroresponse (75th percentile fold increase)	263 (24.2)
Missing data	8 (0.7)

Note: *Al(OH)*_*3*_ - Aluminium hydroxide; *CDI* - Clostridioides difficile infection; *IgG* - Immunoglobulin G; *IQR* - Interquartile range; *ELISA* - Enzyme-linked immunosorbent assay (ELISA); *TNA* - Toxin neutralization assay; *CCI* - Charlson comorbidity index (modified). *Including participants from Latin America and the Asia-Pacific region.

### Association of baseline variables with seroresponse outcomes

**Fig. 2:**
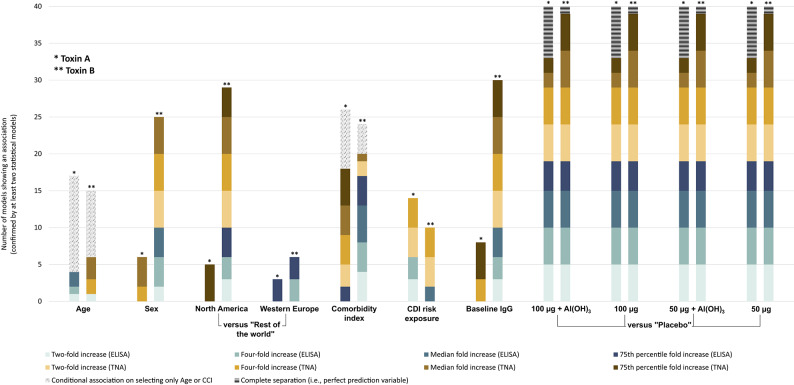
Confirmed model associations (by at least two statistical models) between the different predictors and seroresponse outcomes. Note:* Al(OH)*_*3*_ - Aluminium hydroxide; *CDI* - Clostridioides difficile infection; *IgG* - Immunoglobulin G; *ELISA* - Enzyme-linked immunosorbent assay (ELISA); *TNA* - Toxin neutralization assay; *Comorbidity index (CCI)* - Charlson comorbidity index (modified).

In addition to the vaccines as prime predictors, sex, baseline IgG level and study region (specifically North America) were often found to be associated with both ELISA and TNA seroresponse for TcdB, whereas for TcdA associations were observed mainly with the TNA seroresponse (Fig. 2; Tables 2 and 3). A medium correlation (ρ > 0.7) was observed between age and comorbidity index and therefore they were included mutually exclusively in the logistic regression models and the mixed-effect models as these models cannot appropriately handle collinearity. The comorbidity index showed more consistent associations with seroresponse for various models and settings compared to age. Additionally, future CDI risk exposure, defined as impending hospitalization or nursing home admission within 60 days, compared to prior hospitalizations and systemic antibiotic use within the past year, was primarily associated with two- and four-fold seroresponse to both TcdA and TcdB.

**Table 2:** Predictors of seroresponse against *C. difficile* Toxin A.

Variable	Seroresponse - Toxin A (ELISA)				Seroresponse - Toxin A (TNA)			
	Two-fold increase	Four-fold increase	Median fold increase	75th percentile fold increase	Two-fold increase	Four-fold increase	Median fold increase	75th percentile fold increase
Age (> 65 years)	LR*+SLR	LR*+SLR + MEM*	LR*+SLR + MEM* *+BOOST*	LR*+MEM*	LR*+MEM*	LR*+MEM*	LR*+MEM*	–
Sex (female)	–	–	–	*BOOST*	–	*LR + SLR*	**LR + SLR+MEM** *+BOOST*	*BOOST*
Study region (versus others)								
North America	–	–	–	–	*BOOST*	*BOOST*	–	*LR + SLR* **+MEM** *+CART+BOOST*
Western Europe	–	–	–	*LR + SLR+MEM*	–	–	–	*CART*
CCI (continuous)	LR*	LR*+MEM*	LR*+MEM*	LR*+SLR + MEM* *+BOOST*	*LR +* SLR+MEM* *+BOOST*	**LR + SLR+MEM** *+BOOST*	**LR + SLR+MEM** **+BOOST**	*LR + SLR+MEM* *+CART+BOOST*
CDI risk exposure (future exposure)	*LR* **+ SLR** *+BOOST*	**LR + SLR** **+BOOST**	–	*BOOST*	**LR + SLR** *+MEM* **+BOOST**	**LR + SLR** *+MEM* **+BOOST**	-	*CART*
Baseline IgG (above median)	–	-	–	–	–	*LR + SLR+MEM*	*BOOST*	**LR + SLR+MEM** *+CART+BOOST*
Vaccine group (versus placebo)								
100 µg + Al(OH)_3_	**LR + SLR+MEM** **+CART+BOOST**	**LR + SLR+MEM** **+CART+BOOST**	**LR + SLR+MEM** **+CART+BOOST**	**LR + SLR+MEM** **+BOOST**	**LR + SLR+MEM** **+CART+BOOST**	**LR + SLR+MEM** **+CART+BOOST**	**CART+BOOST**	**CART+BOOST**
100 µg	**LR + SLR+MEM** **+CART+BOOST**	**LR + SLR+MEM** **+CART+BOOST**	**LR + SLR+MEM** **+CART+BOOST**	**LR + SLR+MEM** *+BOOST*	**LR + SLR+MEM** **+CART+BOOST**	**LR + SLR+MEM** **+CART+BOOST**	**CART+BOOST**	**CART** *+BOOST*
50 µg + Al(OH)_3_	**LR + SLR+MEM** **+CART+BOOST**	**LR + SLR+MEM** **+CART+BOOST**	**LR + SLR+MEM** **+CART+BOOST**	**LR + SLR+MEM** *+BOOST*	**LR + SLR+MEM** **+CART+BOOST**	**LR + SLR+MEM** **+CART+BOOST**	**CART+BOOST**	**CART** *+BOOST*
50 µg	**LR + SLR+MEM** **+CART+BOOST**	**LR + SLR+MEM** **+CART+BOOST**	**LR + SLR+MEM** **+CART+BOOST**	**LR + SLR+MEM** *+BOOST*	**LR + SLR+MEM** **+CART+BOOST**	**LR + SLR+MEM** **+CART+BOOST**	**CART+BOOST**	**CART** *+BOOST*

Note: *Al(OH)*_*3*_ - Aluminium hydroxide; *BOOST* - Boosting model; *CART* - Classification and regression tree model; *CCI*-Charlson comorbidity index (modified); *C. difficile* - Clostridioides difficile; *CDI* - Clostridioides difficile infection; *ELISA* - Enzyme-linked immunosorbent assay (ELISA); *IgG* - Immunoglobulin G; *LR* - Logistic regression model; *MEM* - Mixed-effect model; *SLR* - Stepwise logistic regression model; *TNA* - Toxin neutralization assay.

The LR, SLR, and MEM model abbreviations are presented in the table if the respective variable is associated with a p-value < 0.05 (italics) or < 0.01 (bold).

The CART model abbreviations are presented in the table if the respective variable is contributing with a mean impurity decrease > 0.001 (italics) or > 10 (bold).

The BOOST model abbreviations are presented in the table if the respective variable is contributing with an in-bag risk reduction > 0.001 (italics) or > 0.01 (bold).

*Due to collinearity, the presented association is conditional on selecting only Age or CCI in each of the LR or MEM multivariable models.

A hyphen (-) indicates that no association was found in any of the models.

**Table 3:** Predictors of seroresponse against *C. difficile* Toxin B.

Variable	Seroresponse - Toxin B (ELISA)				Seroresponse - Toxin B (TNA)			
	Two-fold increase	Four-fold increase	Median fold increase	75th percentile fold increase	Two-fold increase	Four-fold increase	Median fold increase	75th percentile fold increase
Age (> 65 years)	LR*+MEM* *+BOOST*	LR*+MEM*	–	–	LR*	LR*+SLR + MEM* *+CART*	LR*+SLR + MEM* *+CART+BOOST*	–
Sex (female)	*LR + SLR*	*LR + SLR+MEM* *+BOOST*	*LR + SLR* **+MEM** *+BOOST*	–	**LR + SLR+MEM** *+CART+BOOST*	**LR + SLR+MEM** *+CART+BOOST*	**LR + SLR+MEM** *+CART+BOOST*	*BOOST*
Study region (versus others)								
North America	*LR* **+ SLR** **+BOOST**	**LR + SLR** **+BOOST**	–	*LR* **+ SLR+MEM** *+BOOST*	**LR + SLR+MEM** **+CART+BOOST**	**LR + SLR+MEM** **+CART+BOOST**	**LR + SLR+MEM** **+CART+BOOST**	**LR + SLR+MEM** *+BOOST*
Western Europe	–	*LR + SLR+MEM*	–	*LR + SLR+MEM*	**CART**	**CART**	**CART**	–
CCI (continuous)	**LR + SLR+MEM** *+BOOST*	**LR + SLR+MEM** *+BOOST*	**LR + SLR+MEM** *+CART+BOOST*	*LR + SLR+MEM* *+BOOST*	LR*+SLR + MEM* *+CART*	*CART*	LR*+MEM* *+CART*	–
CDI risk exposure (future exposure)	*BOOST*	**BOOST**	**LR + SLR**	–	**LR + SLR** **+CART+BOOST**	**LR + SLR** *+CART* **+BOOST**	*CART*	–
Baseline IgG (above median)	*LR + SLR+MEM*	*LR + SLR* **+MEM**	**LR + SLR+MEM** *+BOOST*	–	**LR + SLR+MEM** **+CART+BOOST**	**LR + SLR+MEM** **+CART+BOOST**	**LR + SLR+MEM** **+CART+BOOST**	**LR + SLR+MEM** **+CART+BOOST**
Vaccine group (versus placebo)								
100 µg + Al(OH)_3_	**LR + SLR+MEM** **+CART+BOOST**	**LR + SLR+MEM** **+CART+BOOST**	**LR + SLR+MEM** **+CART+BOOST**	**LR + SLR+MEM** **+BOOST**	**LR + SLR+MEM** **+CART+BOOST**	**LR + SLR+MEM** **+CART+BOOST**	**LR + SLR+MEM** **+CART+BOOST**	**LR + SLR+MEM** **+CART+BOOST**
100 µg	**LR + SLR+MEM** **+CART+BOOST**	**LR + SLR+MEM** **+CART+BOOST**	**LR + SLR+MEM** **+CART+BOOST**	**LR + SLR+MEM** *+BOOST*	**LR + SLR+MEM** **+CART+BOOST**	**LR + SLR+MEM** **+CART+BOOST**	**LR + SLR+MEM** **+CART+BOOST**	**LR + SLR+MEM** **+CART** *+BOOST*
50 µg + Al(OH)_3_	**LR + SLR+MEM** **+CART+BOOST**	**LR + SLR+MEM** **+CART+BOOST**	**LR + SLR+MEM** **+CART+BOOST**	**LR + SLR+MEM** *+BOOST*	**LR + SLR+MEM** **+CART+BOOST**	**LR + SLR+MEM** **+CART+BOOST**	**LR + SLR+MEM** **+CART** *+BOOST*	**LR + SLR+MEM** **+CART** *+BOOST*
50 µg	**LR + SLR+MEM** **+CART+BOOST**	**LR + SLR+MEM** **+CART+BOOST**	**LR + SLR+MEM** **+CART+BOOST**	**LR + SLR+MEM** *+BOOST*	**LR + SLR+MEM** **+CART** *+BOOST*	**LR + SLR+MEM** **+CART** *+BOOST*	**LR + SLR+MEM** **+CART** *+BOOST*	**LR + SLR+MEM** **+CART** *+BOOST*

Note: *Al(OH)*_*3*_ - Aluminium hydroxide; *BOOST* - Boosting model; *CART* - Classification and regression tree model; *CCI* - Charlson comorbidity index (modified); *C. difficile* - Clostridioides difficile; *CDI* - Clostridioides difficile infection; *ELISA* - Enzyme-linked immunosorbent assay (ELISA); *IgG* - Immunoglobulin G; *LR* - Logistic regression model; *MEM* - Mixed-effect model; *SLR* - Stepwise logistic regression model; *TNA* - Toxin neutralization assay.

The LR, SLR, and MEM model abbreviations are presented in the table if the respective variable is associated with a p-value < 0.05 (italics) or < 0.01 (bold).

The CART model abbreviations are presented in the table if the respective variable is contributing with a mean impurity decrease > 0.001 (italics) or > 10 (bold).

The BOOST model abbreviations are presented in the table if the respective variable is contributing with an in-bag risk reduction > 0.001 (italics) or > 0.01 (bold).

*Due to collinearity, the presented association is conditional on selecting only Age or CCI in each of the LR or MEM multivariable models.

A hyphen (-) indicates that no association was found in any of the models.

When evaluating the effect size of the baseline predictors, summarized as a range of odds ratios (ORs) across the different models and outcomes, certain trends could be observed. Specifically, age over 65 years (ORs 0.5–0.7) and an increased comorbidity index (ORs 0.7–0.9 per unit increase) were associated with lower likelihood of seroresponse. On the contrary, factors such as female sex (ORs 1.4–2.1), North American study region (ORs 1.7–5.6), future CDI risk exposure (ORs 1.7–4.4), and high baseline IgG levels (ORs 1.6–2.5) showed higher likelihood for the seroresponse outcomes. Some inconsistencies were also observed, particularly with the baseline IgG variable, which demonstrated greater variability in odds ratios for TNA tests of TcdB. Additionally, while future CDI risk was generally associated with higher immunogenicity, this association did not correspond for TcdB levels measured by the median fold increase in the ELISA test. An overview of the odds ratios ranges is provided in Supplementary Tables 3, and detailed results are provided in the Supplementary Tables 4–11.

**Fig. 3:**
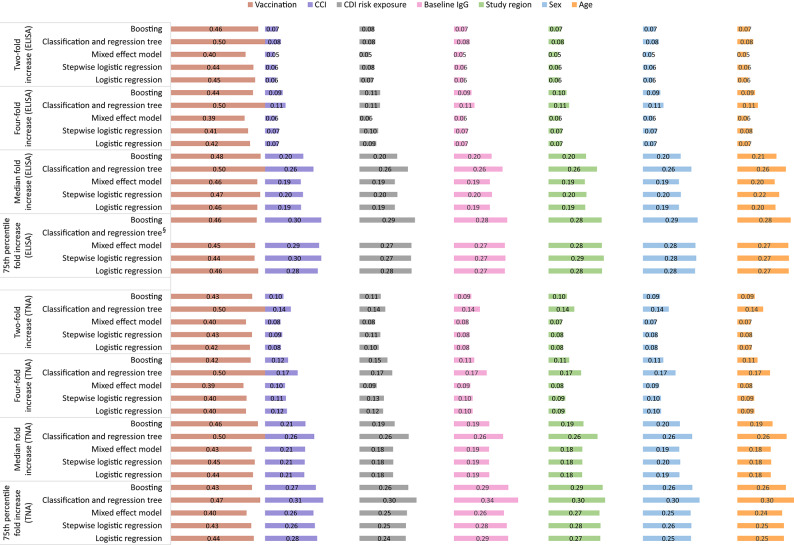
Permutation variable importance for Toxin A defined as 1-AUC (ordered by overall sum across all models). Note:* AUC* - Area under the curve; *CCI* - Charlson comorbidity index (modified); *ELISA* - Enzyme-linked immunosorbent assay (ELISA); *IgG* – Immunoglobulin; *TNA* - Toxin neutralization assay. § - Model convergence issues due to complete separation (perfect prediction).

**Fig. 4:**
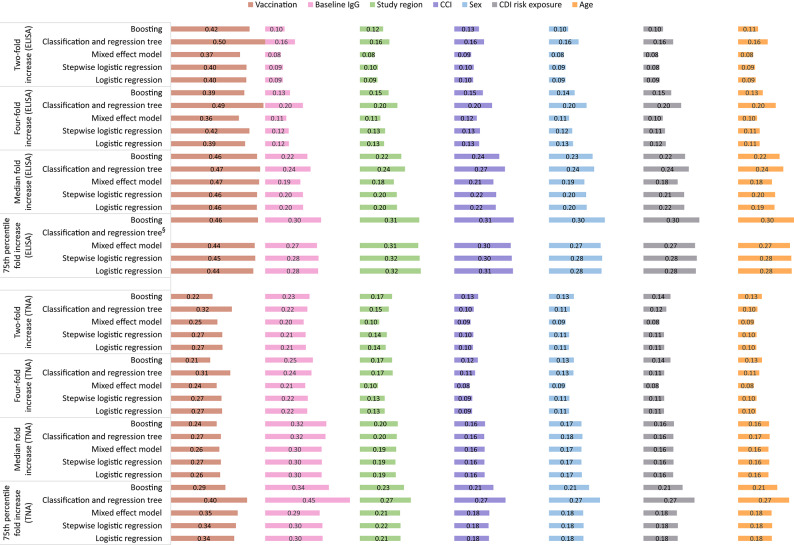
Permutation variable importance for Toxin B defined as 1-AUC (ordered by overall sum across all models). Note:* AUC* - Area under the curve; *CCI* - Charlson comorbidity index (modified); *ELISA* - Enzyme-linked immunosorbent assay (ELISA); *IgG* - Immunoglobulin; *TNA* - Toxin neutralization assay. § - Model convergence issues due to complete separation (perfect prediction).

The results of the model-agnostic variable importance analyses (see Figs. 3 and 4) revealed that predictors beyond the vaccination had a more significant role in models with a median and 75th percentile fold increase seroresponse, compared to models with a two-fold or four-fold increase. Across the models, the variable importance for TcdA was dominated by the vaccination, followed by close relevance of the comorbidity index, CDI risk exposure, baseline IgG, study region, sex, and age. For TcdB, the following order was observed: vaccination, baseline IgG, study region, comorbidity index, sex, CDI risk exposure, and age.

Across all outcome categories, the statistical models showed similar discrimination ability (Tables 4 and 5), with a high score above 0.9 for TcdA and around 0.9 for TcdB, for both two-fold and four-fold seroresponse outcomes. For the median fold increase seroresponse, the AUC measures ranged within a very good score of around 0.8 to 0.85. However, the AUC values were lower for the 75th percentile seroresponse, ranging from 0.7 to 0.8, which can be attributed to the imbalanced outcome categorization, where the models’ ability to distinguish between the participants in this category have been further restrained. Notably, the CART models exhibited lower AUC values in comparison to the other four models. On the contrary, the boosting models showed promising performance, particularly in situations with limited outcomes and high collinearity among variables. This is expected given the characteristics of this model class (see, e.g., Hofner et al. 2012^46^).

**Table 4:** Model performance by the area under the curve.

	Seroresponse - Toxin A (ELISA)				Seroresponse - Toxin A (TNA)			
	Two-fold increase	Four-fold increase	Median fold increase	75th percentile fold increase	Two-fold increase	Four-fold increase	Median fold increase	75th percentile fold increase
Logistic regression	0.946	0.925	0.796	0.694	0.933	0.908	0.842	0.750
Stepwise logistic regression	0.941	0.929	0.796	0.699	0.933	0.905	0.842	0.751
Mixed-effects model	0.942	0.925	0.796	0.694	0.938	0.914	0.842	0.738
Classification and regression tree	0.926	0.914	0.743	§	0.890	0.833	0.767	0.695
Boosting model	0.947	0.928	0.799	0.695	0.921	0.894	0.838	0.733

Note: *ELISA* - Enzyme-linked immunosorbent assay (ELISA); *IgG* - Immunoglobulin G; *TNA* - Toxin neutralization assay.

§ - Model convergence issues due to complete separation (perfect prediction).

**Table 5:** Model performance by the area under the curve.

	Seroresponse - Toxin B (ELISA)				Seroresponse - Toxin B (TNA)			
	Two-fold increase	Four-fold increase	Median fold increase	75th percentile fold increase	Two-fold increase	Four-fold increase	Median fold increase	75th percentile fold increase
Logistic regression	0.887	0.892	0.808	0.714	0.896	0.893	0.821	0.807
Stepwise logistic regression	0.884	0.884	0.808	0.712	0.896	0.891	0.823	0.811
Mixed-effects model	0.893	0.891	0.823	0.728	0.903	0.905	0.821	0.815
Classification and regression tree	0.834	0.807	0.793	§	0.896	0.859	0.822	0.754
Boosting model	0.867	0.880	0.841	0.731	0.886	0.878	0.824	0.825

Note: *ELISA* - Enzyme-linked immunosorbent assay (ELISA); *IgG* - Immunoglobulin G; *TNA* - Toxin neutralization assay.

§ - Model convergence issues due to complete separation (perfect prediction).

## Discussion

We performed multiple analyses on a pooled dataset from two *C. difficile* vaccine clinical trials from Sanofi, to assess the baseline factors associated with different seroresponse outcomes, in regards to TcdA and TcdB, as measured by ELISA or TNA. Across the settings, in addition to the immunization, higher likelihood of seroresponse was associated with lower comorbidity index, age < 65 years, and future CDI risk exposure, with comparable importance for both TcdA and TcdB. In addition, higher baseline IgG, North American study region, and female sex were also shown as relevant predictors, particularly for the seroresponse of TcdB.

The recruitment and retention of the target populations in vaccine trials is a complex challenge in the development process^48^. In contrast to conventional trials that focus on therapeutic interventions for affected patients, vaccine trials involve healthy volunteers and often require larger sample sizes and extended follow-up periods. A comprehensive approach with prior research, stakeholder engagement, and addressing concerns related to potential risks is essential for encouraging heterogenous participation in clinical trials. This is particularly important for risk populations such as elderly and vulnerable subjects, who play a crucial role in determining vaccine effectiveness and are often given priority for vaccination after licensure. Ultimately, this leads to the development of safe and effective vaccines for the general public.^49,50^ Extensive evidence on *C. difficile* highlights the complex interplay of risk factors, including medication (e.g., antibiotics, antiphlogistics, proton-pump inhibitors), host-related factors (e.g., increased age, diabetes mellitus, congestive heart disease) as well as the role of clinical interventions (e.g., surgery, longer hospital stay), in increasing susceptibility to CDI^51,52^. The Sanofi Phase III trial focused on adult participants aged 50 or older who were at an increased risk of CDI due to previous hospitalizations and systemic antibiotic use (risk stratum 1) or a scheduled hospital stay (risk stratum 2). The Phase III trial by Pfizer focused on a wider community-based population of individuals aged 50 years or older who were identified as at risk of CDI due to various factors such as nursing home residency, frequent healthcare visits, or recent antibiotic prescription, without stratification based on prior or future risk exposure^30,31^. Our analyses showed that selecting trial participants with future CDI risk exposure (i.e., impending hospitalization or long-term care/rehabilitation facility stay) could result with higher immunogenicity, in comparison to the participants with past risk exposure (i.e., risk stratum 1). Still, it is important to further evaluate the representation of these subcategories and to consider the influence of the different time-lapse between past and predicted future hospitalization. The observed results might also reflect that the immune response to *C. difficile* is boosted in individuals hospitalized after vaccination once they become naturally exposed, whereas those hospitalized before vaccination may not have acquired and maintained sufficiently high immunity, leading to a weaker response to the vaccination, which may, however, also result from immunocompromised states. Other potential confounders, including the interactions among frailty, underlying comorbidities, and immunosenescence could also influence the immunogenicity and contribute to the observed results, and should be further investigated.

Immunosenescence is a well-known phenomenon in which immune defense declines with age, making the elderly more susceptible to infections and also less capable to reach an effective immune response to vaccines^53,54^. Comorbidities, or existing medical conditions, contribute to this decline. Previous studies demonstrated an association between increased age and comorbidities on vaccine effectiveness^55,56^. Several published studies on *C. difficile* vaccines examined their ability to induce an immune response in individuals of different age groups^57,58^. In Sanofi’s Phase I studies, 50 healthy adults (18–55 years) and 48 elderly subjects (≥ 65 years) were randomized to receive a three-dose toxoid vaccine (with single doses either 2, 10, or 50 µg with Al(OH)_3_), or placebo. Study results showed that older individuals had a lower geometric mean concentration and showed slower and weaker seroresponse (i.e., four-fold increase from baseline, measured by ELISA) compared to younger individuals^59^. Follow-up studies focused on a specific age-range (e.g., 40–75 years or > 50 years), and also assessed the ELISA and TNA results as well as a composite immunogenicity outcome defined as (at least) four-fold increase for both toxins at the same time^30,40^. In a Phase I trial of Valneva, evaluating the three-dose immunization with VLA84 (20 µg, 70 µg, and 200 µg with/without Al(OH)_3_), 60 healthy adults (18–64 years) and 80 elderly subjects (≥ 65 years) were enrolled in the study. Immunogenicity was assessed using ELISA and TNA results (geometric mean concentration/titers and composite seropositivity with more than four-fold increase from baseline against TcdA and TcdB simultaneously). Results showed that middle-aged adults had a faster immune response compared to the elderly group, but both populations had similar levels of IgG antibodies at their peak^32^. On the other hand, a Phase I trial evaluating Pfizer’s three-dose toxoid vaccine (with single doses either 50, 100, or 200 µg with/without Al(OH)_3_; Placebo), including 97 adults (50–64 years old) and 95 elderly subjects (65–85 years old) showed comparable immunological response levels in both age groups^60^. The immunogenicity was measured based on the TNA neutralizing antibody levels, comparing the geometric mean concentration and geometric mean fold ratios. Follow-up studies focused on specific age-range (e.g., 65–85 years or > 50 years), and also assessed the 4-, 8-, 16-, and 32-fold increases from baseline at various time points, along with a newly developed neutralizing threshold^31,61–63^. Immunogenicity results from the Phase III trial are still pending. While the studies differ in multiple factors (such as vaccine formulation, dosage, and timing), which makes direct comparisons challenging, the *C. difficile* vaccine trials generally suggest inconsistent correlations between age and immunogenicity, as well as a lack of understanding regarding the influence of comorbidities. Furthermore, there are variations in the methods used to assess immunogenicity between different studies and vaccines. Our findings demonstrate a reverse association between the modified Charlson comorbidity index (accounting for age categories) and the seroresponse outcomes. This implies that an increased comorbidity index is associated with a lower likelihood of a strong immune response, which could support the vaccine development, particularly for preventing CDI, where true levels of protection have not yet been established, and which is mainly targeting the elderly. Therefore, it is essential to thoroughly assess and compare comorbidities and age versus the immunological response in order to improve comparability across the studies and gain a better understanding of the vaccine response.

A further relevant factor of seroresponse particularly for the TcdB neutralizing capacity was a higher baseline IgG. Many healthy adults (∼60%) have detectable serum IgG and IgA in regards to TcdA and TcdB, which could be a result of childhood exposure or sub-clinical infections^64,65^. Baseline seropositivity was correlated with significantly elevated immune reactions in the Phase II trial by Pfizer, particularly for TcdB^61^, and baseline values above the lower limit of quantification showed stronger immune responses. This was also noted in the Phase I trial by GlaxoSmithKline^34^. Furthermore, in Valneva’s Phase I trial^32^ non-adjuvanted vaccine formulations were as immunogenic as adjuvanted ones, unlike the findings from the preclinical studies with naïve mice. This suggests that individuals with detectable levels of serum IgG against TcdA and TcdB may have a more robust immune response after immunization, likely due to pre-existing memory B cells, highlighting the importance of effective vaccination, particularly for older, at-risk individuals or those with lower baseline antibody levels. Still, the lack of alignment between the immune responses such as serum neutralizing or non-neutralizing antibodies to TcdA or TcdB and the clinical protection in *C. difficile* vaccine studies highlights the need for validated immune correlates of protection.

The differences that we observed in terms of the study regions (especially a higher immune response in North America) are likely reflecting the geographic variation of *C. difficile* ribotypes and toxin gene variants, leading to genetic diversity in toxin structure, expression and epitope representation. Since humoral immunity is usually specific to encountered toxin variants, vaccines or mAbs may not protect against all circulating strains, risking failure if novel variants appear. This challenge has halted vaccine development for many pathogens and cannot be ruled out for the currently analyzed *C. difficile* vaccine. Still, a recent analysis of the MODIFY trial samples^66^ showed that North America has the highest diversity and representation of clades and ribotypes, supporting the proposed region for a *C. difficile* vaccine assessment. However, bias in the analyses due to underrepresentation of the global south, as well as the risk of trial failure from insufficient strain coverage, cannot be excluded. Recent work by Mahool et al.^67^ describes a similar phenomenon by comparing strain coverage of two mAbs directed at TcdB. The sequence comparisons highlight the risk of insufficient representation of the epitope recognized by mAbs in infecting strains and, in this case, the superiority in regards to coverage of a new mAb developed by AstraZeneca when compared to the licensed product (Bezlotoxumab) from Merck. Given the high toxin diversity, a thorough assessment of ribotype as well as toxin variant and sequence type is recomended^68^. In addition to differences in *C. difficile* strains, regional variation may also be influenced by human genetic factors affecting the immune function, with possible enhancements due to socioeconomic and race/ethnic disparities. For example, since individuals from lower socioeconomic or specific racial/ethnic backgrounds experience increased immunosenescence due to genetic and socio-environmental factors, the data may be influenced by the concentration of trial participants on those from North America with higher financial means (e.g., living in a nursing home or with elective surgeries).^69,70^ Overall, the diverse genetic backgrounds and environmental exposures of participants in a study should motivate wider enrolment efforts and multi-regional trials, aiming for a diverse and representative sample among the treatment arms.

Sex differences play a significant role in shaping both the innate and adaptive immune responses. Environmental factors, including nutrition status and the composition of the microbiome, also alter the development and functioning of the immune system differently in males and females, resulting in sex-specific immune responses that can impact susceptibility to autoimmune diseases, malignancies, and infectious diseases, as well as affect the outcome of vaccination^71^. The effectiveness of vaccines that are specifically recommended for older adults has been consistently observed to be higher among females as compared to males^72^, which is also in line with the findings from our analyses.

Our study has several limitations. Dichotomizing the outcome bears the risk of losing information and may result in underestimating variations or masking nonlinear relationships between the predictors and seroresponse. In contrast, the dichotomization may help interpret and understand the data in regards to our study aim, which was to identify relevant predictors of a robust immune response. Historically, a two-fold increase in antibody levels could be attributed to inherent assay variability rather than the vaccination itself, and therefore the four-fold increase was seen as a more reliable marker of seroresponse^73^. To provide a more comprehensive assessment, we assessed both two-fold and four-fold increases, as well as the median and 75th percentile fold increases for the specific study group. This approach offered a more holistic evaluation of immune response, allowing us to better assess the specific population exhibiting strong immune responses, and to explore the changes in the variable importance across the different outcome settings. Still, this approach is based on the hypothesis that the vaccines booster the preexisting B cell memory and antibody response. It may not be valid if a new pool of memory B cells is induced from naïve B cells by immunization.

We restricted the analyses to study participants who received three doses of the vaccine and were compliant with the protocol. This allowed for better comparability in the pooled dataset but might not be representative for the setting in which patients received different numbers of doses. Also, we did not account for different vaccine schedules due to the limited cases compared to the predominant 0, 7, 30-day schedule. To address potential confounding from the different vaccine schedules in the Phase II trial, we conducted a sensitivity analysis and excluded 118 participants who received the three-dose vaccine on 0, 7, 180 or 0, 30, 180 days. We observed a reduction in the number of significant associations, particularly for Toxin A (where associations decreased from 182 to 152), a decline in the strength of statistical associations (e.g., p-values shifting from < 0.001 to < 0.05), and higher number of models showing complete separation (perfect prediction) for the 75th percentile fold increase outcomes. This decline in the number of associations mainly affected the study region variable, with the Western Europe region becoming less relevant, and also impacted the variable age and Northern America in regards to TcdA (Supplementary Tables 14–15). These changes may be due to the differences between the vaccine regimens and due to the smaller dataset. However, we observed an overall similar pattern to the main analyses.

Despite efforts to increase the interpretability of the models by considering different levels of statistical significance and variable importance, variations in model characteristics and outputs hindered the comparisons and occasionally lead to sporadic associations. Therefore, we also considered a model-agnostic approach for comparing the variable importance of the predictors. Due to limited data, we could not directly assess other potential predictors of immunogenicity, such as social determinants of health (e.g., socioeconomic status, healthcare access, living conditions), behavioral and nutritional factors (e.g., exercise, alcohol consumption, smoking, nutritional status, body mass index, stress level), as well as key factors like genetics and microbiome status.

Lastly, while we used multivariable analyses and advanced methods to estimate the effects of the predictor on seroresponse and provide solid foundation for further research, our analysis was intended to highlight potential associations rather than demonstrating causal relationships. Assessing specific causal effects would require a focused research question and different methodological approaches, such as g-methods^74^. Therefore, these results should not be considered as evidence of causality.

In conclusion, developing an effective vaccine for *C. difficile* poses numerous challenges, including the anticipation of individuals who will be most likely affected and of the time frame in which they will develop symptoms. By identifying baseline clinical characteristics and pre-vaccination immune signatures (e.g., genomic differences), one could better predict the vaccine response, implement tailored vaccination strategies (i.e., personalized or precision vaccination), and increase the vaccine safety and success rate^75^. Our analyses showed that lower comorbidity index, age < 65 years, future CDI risk exposure, higher baseline IgG, North American study region, and female sex were relevant predictors of seroresponse to a *C. difficile* vaccine candidate. Considering the different outcomes, modeling approaches, and imposed data limitations, the analyses appeared to be largely consistent in terms of the effect size of the predictors. Improved clinical trial planning, stratified evaluation, as well as the possibility to optimize safety and efficacy by accounting for appropriate immunological protection and adjusting the doses, dose schedules, or vaccine components based on the different predispositions to achieve high immunogenicity should be further assessed. Last but not least, our study aligns with recent evidence highlighting challenges in data accessibility^76^ and shows that wider knowledge generation through enhanced collaboration, data sharing and analyses across clinical trials (hopefully also from different vaccine developers in the future) is needed to tackle upcoming, dire challenges such as the antimicrobial resistance.

## Supplementary Information

Below is the link to the electronic supplementary material.

Supplementary Material 1

## Data Availability

Data can be obtained through the Vivli platform (www.vivli.org) in agreement with Sanofi. All analysis results and analysis code are available from the corresponding author upon reasonable request.
