# Supplementary material for: Pooled analyses of Clostridioides difficile vaccine trials identify baseline predictors for vaccine response
Source: Sci Rep. 2026 Mar 12;16:8981. doi: 10.1038/s41598-026-42375-5 (PMC12992666; doi:10.1038/s41598-026-42375-5)
Supplement: Supplementary file 1 — Supplementary Material 1 [file 41598_2026_42375_MOESM1_ESM.docx]

**Supplementary information**

*Data structure and analyses*

We made several assumptions in the data structure in order to conduct a comprehensive analysis of the pooled data from the two studies. The variable “Age” was provided as an ordinal variable, with varying ranges for each trial (i.e., 40.15-55.56, 55.56-64.93, 64.93-69.34, and 69.34-75.97 for the phase II trial, and 49.02-58.76, 58.76-65.54, 65.54-72.07, and 72.07-98.26 for the phase III trial). To standardize the “Age” variable, a cut-off point of 65.5 years was used to create two categories (i.e., age below 65.5 years and age above 65.5 years). The variable “Sex” was consistent across all two studies, while in the “Study region” variable we combined the “North America” with the “USA” observations, and the seven observations of “Eastern Europe” with “Western Europe”. The sub-category "Rest of the world" was used as provided, likely compiling participants from Latin America and the Asia-Pacific region. The difference in our analyses were observed despite the stratified randomization by geographic region in the Phase III trial. However, it should also be acknowledged that for our immunogenicity analyses the North American region was dominant in comparison to the “Rest of the world" sub-category, which likely encompasses the Latin America and Asia-Pacific regions. Furthermore, we defined the variable "CDI risk exposure" as either future or past risk. Future risk included participants from the Phase II trial with impending hospitalization or long-term care/rehabilitation facility stay within 60 days, combined with Phase III risk stratum 2 participants (anticipated hospital admission ≥72 hours for elective surgery within 60 days). Past risk was defined by Phase III risk stratum 1 participants, who had at least two prior hospitalizations (≥24 hours each) and systemic antibiotic exposure within the previous 12 months. The variable “Vaccine group” was also combined, grouping data based on overlapping dosing and vaccine composition. Differences in vaccine schedule were not considered, as the focus was on immunogenicity outcomes within 30 days after the last vaccine, regardless of the specific vaccine schedule used. The variables “Body mass index” and “Race” were excluded from the analyses due to incomplete data and heterogeneous coding, making it difficult to find common overlaps. Additionally, a modified “Charslon comorbidity index” was developed as a robust prognostic and health risk stratification tool to evaluate the comorbidity burden among study participants. As the data did not contain International Classification of Diseases coding, we performed a manual search for overlapping diagnoses using the categories and scoring system presented in Supplementary Table 14. This scoring system was informed by previous research conducted by Quan et al.^1^ and Glasheen et al.^2^, including 19 categories with assigned weights. It should be noted that since we did not have access to the level of granularity required for this score, but only to a less detailed one, we adopted a conservative approach and used lower weights whenever we could not clearly distinguish disease stages that required different weights (e.g., diabetes with or without chronic complications). We assume that this will not have serious impact on the overall reliability, as it is likely that severely ill individuals would not meet the trial inclusion criteria. Furthermore, possible hierarchical categories for participants were taken into consideration, where only the weights from the more advanced stage were utilized in case of co-existence of moderate-severe or lower-milder stages. The final score was obtained by summarizing the individual weights for each of the categories and further including weights for the age category, using 0 points for age 0-49, 1 point for age 50-59, 2 points for age 60-69, 3 points for age 70-79, 4 points for age 80-89, and 5 points for age ≥90. The age of each individual participant, as provided by the age range in each trial, was aligned as closely as possible with the corresponding age-specific weights for calculating the score.

For the analyses, we used five different models to identify the potential predictors of seroresponse outcomes. Before developing these models, we examined possible correlations and discussed overlapping variables. Both Pearson and Spearman correlation analyses informed this preliminary assessment. A medium correlation (ρ > 0.7) was observed between age and comorbidity index and therefore they were included mutually exclusively in the logistic regression models and the mixed-effect models as these models cannot appropriately handle collinearity. Next, we proceeded with the model development. In the logistic regression models, we started with a full, saturated model that included all baseline predictors, considering age and comorbidity index separately. For our stepwise logistic regression models, we employed the Akaike Information Criterion to determine the best-fitting model, by assessing the trade-off between the goodness of fit and the complexity of the model, favoring simpler models that could adequately explain the data. For the mixed-effects model, we incorporated the trial (i.e., Phase II or Phase III) as a random effect. This approach could help in the control for differences that might influence the outcomes across the trial settings. In the classification and regression tree analyses, we used the default setting with ten-fold cross-validation and complexity parameter of 0.01, which controls the extent of tree pruning by eliminating splits that do not enhance the model's fit by at least this factor. For developing the boosting models, we determined the optimal number of boosting steps (mstop) for each model by using 25-fold bootstrap cross-validation, thereby minimizing the cross-validated predictive risk, balancing model complexity and generalization. Overall, vaccination was part of all models due to the expected high relevance for the outcomes. All models were developed using 75% of the pooled data as a training set and the remaining 25% as a test set. For the analyses we used the following R packages: stats^3^, MASS^4^, lme4^5^, rpart^6^, mboost^7^, pROC^8^, caret^9^, sjPlot^10^, and Table1^11^.

Supplementary Table 1. Median cut-off points used for defining the baseline IgG variables

|  | Phase II trial  Median [IQR] | Phase III trial  Median [IQR] |
| --- | --- | --- |
| Baseline IgG-ELISA (EU/mL) |  |  |
| Toxin A | 0.75 [0.75-0.75] | 0.75 [0.75-0.75] |
| Toxin B | 0.40 [0.40-3.30] | 1.20 [0.40-2.60] |
| Baseline IgG-TNA (1/dil) |  |  |
| Toxin A | 8.00 [8.00-8.00] | 8.00 [8.00-8.00] |
| Toxin B | 8.00 [8.00-25.0] | 8.00 [8.00-8.00] |

IgG - Immunoglobulin G; IQR - Interquartile range; ELISA - Enzyme-linked immunosorbent assay (ELISA);

EU/ml - Endotoxin units per milliliter; TNA - Toxin neutralization assay.

Supplementary Table 2. Median cut-off points used for defining the seroresponse outcome variables

|  | Phase II trial  Median | Phase III trial  Median |
| --- | --- | --- |
| Outcome IgG-ELISA (60 days) |  |  |
| Toxin A (Median fold increase) | 42 | 12 |
| Toxin A (75^th^ percentile fold increase) | 128 | 45 |
| Toxin B (Median fold increase) | 55 | 3 |
| Toxin B (75^th^ percentile fold increase) | 206 | 30 |
| Outcome IgG-TNA (60 days) |  |  |
| Toxin A (Median fold increase) | 22 | 6 |
| Toxin A (75^th^ percentile fold increase) | 67 | 25 |
| Toxin B (Median fold increase) | 6 | 1 |
| Toxin B (75^th^ percentile fold increase) | 89 | 2 |
| Outcome IgG-ELISA (210 days)* |  |  |
| Toxin A (Median fold increase) | 79 | - |
| Toxin A (75^th^ percentile fold increase) | 239 | - |
| Toxin B (Median fold increase) | 39 | - |
| Toxin B (75^th^ percentile fold increase) | 116 | - |
| Outcome IgG-TNA (210 days)* |  |  |
| Toxin A (Median fold increase) | 176 | - |
| Toxin A (75^th^ percentile fold increase) | 808 | - |
| Toxin B (Median fold increase) | 41 | - |
| Toxin B (75^th^ percentile fold increase) | 185 | - |

IgG - Immunoglobulin G; IQR - Interquartile range; ELISA - Enzyme-linked immunosorbent assay (ELISA);

TNA - Toxin neutralization assay

*In the Phase II trial two additional dosing schedules were explored: 0, 7, 180 days and 0, 30, 180 days.

Supplementary Table 3. Range of observed odds ratios

| Variable | Seroresponse - Toxin A | | Seroresponse - Toxin B | |  |
| --- | --- | --- | --- | --- | --- |
|  | ELISA | TNA | ELISA | TNA | |
| Age  (>65years) | 0.5-0.7 | 0.5-0.6 | 0.3-0.5 | 0.6-0.7 | |
| Sex  (female) | * | 1.4-1.8 | 1.6-1.8 | 1.7-2.1 | |
| Study region  (versus others) |  |  |  |  | |
| North America | * | 1.7-2.2 | 1.8-2.5 | 2.5-5.6 | |
| Western Europe | 2.2-2.3 | * | 2.6-3.1 | * | |
| CCI (continuous) | 0.8-0.9 | 0.7-0.9 | 0.7-0.9 | 0.9-0.9 | |
| CDI risk exposure  (future exposure) | 2.6-3.6 | 1.7-4.4 | 0.5-0.6 | 2.1-2.5 | |
| Baseline IgG  (above median) | * | 2.1-2.5 | 1.6-2.0 | 7.8-49.3 | |
| Vaccine group  (versus placebo) |  |  |  |  | |
| 100 µg + Al(OH)_3_ | 105-13990 | 1651-3313 | 97-259 | 15-305 | |
| 100 µg | 70-1569 | 199-688 | 69-695 | 31-506 | |
| 50 µg + Al(OH)_3_ | 74-1804 | 514-1977 | 56-321 | 19-349 | |
| 50 µg | 38-2177 | 168-634 | 66-274 | 11-190 | |

The presented ranges of odds ratios are summarized across the different seroresponse outcomes and statistical models (i.e., logistic regression (full & stepwise) and mixed effect models

*No associations were observed in the analyses using the proposed models.

Supplementary Table 4.1 Logistic regression multivariable model (full model) – ELISA and TNA measurements (Anti-toxin A IgG)

| Variable | Seroresponse - Toxin A (ELISA) | | | | | | | | | Seroresponse - Toxin A (TNA) | | | | | | | |
| --- | --- | --- | --- | --- | --- | --- | --- | --- | --- | --- | --- | --- | --- | --- | --- | --- | --- |
|  | Two-fold increase | | Four-fold increase | | Median fold increase | | | 75^th^ percentile fold increase | | Two-fold increase | | Four-fold increase | | Median fold increase | | 75^th^ percentile fold increase | |
|  | OR | CI | OR | CI | OR | CI | OR | | CI | OR | CI | OR | CI | OR | CI | OR | CI |
| Age  (>65 years) | 0.67 | 0.27 –  1.63 | 0.66 | 0.29 –  1.43 | 0.65 | 0.40 –  1.06 | 0.89 | | 0.55 – 1.46 | 0.81 | 0.40 – 1.63 | 1.16 | 0.63 – 2.15 | 1.06 | 0.65 – 1.73 | 1.15 | 0.69 – 1.91 |
| Sex  (female) | 1.12 | 0.58 –  2.17 | 1.37 | 0.77 –  2.49 | 1.24 | 0.87 –  1.76 | 1.32 | | 0.94 – 1.86 | 1.27 | 0.76 – 2.15 | 1.71* | 1.08 – 2.74 | 1.66 ** | 1.17 – 2.36 | 1.30 | 0.92 – 1.85 |
| Study region  (versus others) |  | |  | |  | | |  | |  |  |  |  |  |  |  |  |
| North America | 1.22 | 0.49 –  3.00 | 1.48 | 0.69 –  3.14 | 1.03 | 0.61 –  1.72 | 1.28 | | 0.77 – 2.14 | 1.46 | 0.71 – 2.98 | 1.25 | 0.66 – 2.34 | 0.88 | 0.52 – 1.49 | 1.72 * | 1.02 – 2.95 |
| Western Europe | 2.46 | 0.57 –  12.53 | 2.28 | 0.63 –  12.03 | 2.05 | 0.83 –  5.66 | 2.18* | | 1.01 – 4.72 | 0.91 | 0.34 – 2.71 | 0.91 | 0.36 – 2.43 | 1.16 | 0.51 – 2.82 | 1.08 | 0.43 – 2.51 |
| CCI (continuous) | 0.89 | 0.69 –  1.17 | 0.88 | 0.71 –  1.11 | 0.95 | 0.82 –  1.11 | 0.89 | | 0.76 – 1.04 | 0.82* | 0.67 – 1.00 | 0.73 *** | 0.61 – 0.87 | 0.79 ** | 0.68 – 0.92 | 0.85 * | 0.72 – 0.99 |
| CDI risk exposure  (future exposure) | 2.84* | 1.18 –  7.26 | 2.93** | 1.38 –  6.44 | 1.10 | 0.68 –  1.76 | 1.28 | | 0.82 – 2.01 | 3.47 *** | 1.72 – 7.28 | 3.54 *** | 1.93 – 6.64 | 1.02 | 0.64 – 1.64 | 1.00 | 0.63 – 1.59 |
| Baseline IgG  (above median) | 1.21 | 0.48 –  3.54 | 0.67 | 0.32 –  1.54 | 0.76 | 0.46 –  1.25 | 0.95 | | 0.57 – 1.55 | 2.27 | 0.96 – 6.37 | 2.47* | 1.15 – 6.00 | 1.71 | 1.01 – 3.00 | 2.16 ** | 1.35 – 3.47 |
| Vaccine group  (versus placebo) |  | |  | |  | | |  | |  |  |  |  |  |  |  |  |
| 100 µg + Al(OH)_3_ | 972 *** | 363 –  3310 | 2880 *** | 585 –  52480 | 533 *** | 117 –  9451 | 107 *** | | 24 – 1897 | 2688 *** | 542 –  49100 | 1908 *** | 392 –34595 | § | § | § | § |
| 100 µg | 323 *** | 88 –  1562 | 1566 *** | 244 –  32154 | 303 *** | 58 –  5586 | 71  *** | | 14 – 1304 | 621 *** | 111 –11831 | 519 *** | 95 –9776 | § | § | § | § |
| 50 µg + Al(OH)_3_ | 393 *** | 106 –  1913 | 1798 *** | 281 –  36929 | 220 *** | 43 –  4041 | 74  *** | | 14 – 1366 | 1861 *** | 291 –  38180 | 1374 *** | 231 –26996 | § | § | § | § |
| 50 µg | 462 *** | 115 –  2611 | 2177 *** | 317 –  46887 | 205 *** | 40 –  3783 | 38  *** | | 7 – 711 | 582 *** | 105 –  11079 | 443 *** | 82 –  8331 | § | § | § | § |

* p<0.05; ** p<0.01; *** p<0.001; § - Variable omitted due to complete separation (perfect prediction)

Al(OH)_3_ - Aluminium hydroxide; CCI - Charlson comorbidity index (modified); CDI - Clostridioides difficile infection; CI - Confidence interval; ELISA - Enzyme-linked immunosorbent assay; IgG - Immunoglobulin G; OR - Odds ratio; TNA - Toxin neutralization assay.

Supplementary Table 4.2 Logistic regression multivariable model (alternatively including either age or comorbidity index as independent variable) – ELISA measurements (Anti-toxin A IgG)

| Variable | Seroresponse - Toxin A (ELISA) | | | | | | | | | | | | | | | | |
| --- | --- | --- | --- | --- | --- | --- | --- | --- | --- | --- | --- | --- | --- | --- | --- | --- | --- |
|  | Two-fold increase | | Two-fold increase | | Four-fold increase | | | Four-fold increase | | Median fold increase | | Median fold increase | | 75^th^ percentile fold increase | | 75^th^ percentile fold increase | |
|  | OR | CI | OR | CI | OR | CI | OR | | CI | OR | CI | OR | CI | OR | CI | OR | CI |
| Age  (>65 years) | 0.51 * | 0.26 – 0.99 | - | - | 0.48 * | 0.27 – 0.87 | - | | - | 0.58 ** | 0.41 – 0.82 | - | - | 0.69 * | 0.49 – 0.97 | - | - |
| Sex  (female) | 1.14 | 0.60 – 2.21 | 1.10 | 0.57 – 2.13 | 1.40 | 0.78 – 2.54 | 1.35 | | 0.75 – 2.45 | 1.25 | 0.88 – 1.77 | 1.22 | 0.86 – 1.73 | 1.33 | 0.95 – 1.88 | 1.31 | 0.93 – 1.85 |
| Study region  (versus others) |  | |  | |  | | |  | |  |  |  |  |  |  |  |  |
| North America | 1.12 | 0.46 – 2.71 | 1.28 | 0.52 – 3.15 | 1.34 | 0.64 – 2.78 | 1.56 | | 0.74 – 3.30 | 0.99 | 0.59 – 1.65 | 1.09 | 0.65 – 1.83 | 1.20 | 0.73 – 2.00 | 1.30 | 0.79 – 2.17 |
| Western Europe | 2.30 | 0.55 – 11.50 | 2.50 | 0.58 – 12.83 | 2.16 | 0.60 – 11.26 | 2.31 | | 0.63 – 12.19 | 2.01 | 0.82 – 5.55 | 2.07 | 0.84 – 5.72 | 2.11 | 0.98 – 4.56 | 2.18 * | 1.01 – 4.72 |
| CCI (continuous) | - | - | 0.82 * | 0.68 – 1.00 | - | - | 0.81 * | | 0.69 – 0.96 | - | - | 0.87 ** | 0.78 – 0.96 | - | - | 0.87 * | 0.78 – 0.97 |
| CDI risk exposure  (future exposure) | 3.11 * | 1.32 – 7.79 | 2.63 * | 1.11 – 6.62 | 3.26 ** | 1.57 – 7.02 | 2.69 ** | | 1.29 – 5.83 | 1.14 | 0.71 – 1.80 | 1.01 | 0.63 – 1.60 | 1.38 | 0.89 – 2.14 | 1.25 | 0.81 – 1.95 |
| Baseline IgG  (above median) | 1.22 | 0.48 – 3.54 | 1.25 | 0.49 – 3.65 | 0.68 | 0.32 – 1.56 | 0.69 | | 0.33 – 1.58 | 0.76 | 0.46 – 1.26 | 0.77 | 0.47 – 1.27 | 0.96 | 0.58 – 1.57 | 0.96 | 0.58 – 1.56 |
| Vaccine group  (versus placebo) |  | |  | |  | | |  | |  |  |  |  |  |  |  |  |
| 100 µg + Al(OH)_3_ | 942  *** | 353 –3198 | 980  *** | 367 –3328 | 2782 *** | 566 –50664 | 2894 *** | | 589 –52677 | 529 *** | 116 –9375 | 542  *** | 119 –9604 | 105 *** | 23 – 1860 | 108 *** | 24 –1911 |
| 100 µg | 320 *** | 87 –1549 | 326 *** | 89 –1571 | 1550 *** | 242 –31844 | 1569 *** | | 246 –32180 | 302 *** | 58 –5583 | 308  *** | 60 –5688 | 70  *** | 14 – 1299 | 71  *** | 14 – 1314 |
| 50 µg + Al(OH)_3_ | 394 *** | 106 –1920 | 389  *** | 106 –1884 | 1804 *** | 281 –37089 | 1766 *** | | 277 –36201 | 221  *** | 43 –4060 | 220  *** | 43 –  4041 | 75  *** | 15 – 1377 | 75  *** | 15 –1370 |
| 50 µg | 458  *** | 115 –2592 | 459 *** | 115 –2583 | 2161 *** | 315 –46556 | 2149 *** | | 314 –46202 | 206  *** | 40 –3796 | 207  *** | 40 –3808 | 38  *** | 7 – 718 | 38  *** | 7 – 715 |

* p<0.05; ** p<0.01; *** p<0.001

Al(OH)_3_ - Aluminium hydroxide; CCI - Charlson comorbidity index (modified); CDI - Clostridioides difficile infection; CI - Confidence interval; ELISA - Enzyme-linked immunosorbent assay; IgG - Immunoglobulin G; OR - Odds ratio; TNA - Toxin neutralization assay.

A hyphen (-) indicates that no association was found in any of the models.

Supplementary Table 4.3 Logistic regression multivariable model (alternatively including either age or comorbidity index as independent variable) – TNA measurements (Anti-toxin A IgG)

| Variable | Seroresponse - Toxin A (TNA) | | | | | | | | | | | | | | | | |
| --- | --- | --- | --- | --- | --- | --- | --- | --- | --- | --- | --- | --- | --- | --- | --- | --- | --- |
|  | Two-fold increase | | Two-fold increase | | Four-fold increase | | | Four-fold increase | | Median fold increase | | Median fold increase | | 75^th^ percentile fold increase | | 75^th^ percentile fold increase | |
|  | OR | CI | OR | CI | OR | CI | OR | | CI | OR | CI | OR | CI | OR | CI | OR | CI |
| Age  (>65 years) | 0.50 ** | 0.30 – 0.84 | - | - | 0.56 * | 0.35 – 0.87 | - | | - | 0.62 ** | 0.44 – 0.87 | - | - | 0.79 | 0.56 – 1.12 | - | - |
| Sex  (female) | 1.31 | 0.79 – 2.21 | 1.26 | 0.75 – 2.12 | 1.78 * | 1.13 – 2.83 | 1.73 * | | 1.09 – 2.76 | 1.70 ** | 1.20 – 2.41 | 1.66 ** | 1.17 – 2.37 | 1.32 | 0.93 – 1.88 | 1.31 | 0.92 – 1.86 |
| Study region  (versus others) |  | |  | |  | | |  | |  |  |  |  |  |  |  |  |
| North America | 1.25 | 0.62 – 2.49 | 1.50 | 0.74 – 3.05 | 0.99 | 0.54 – 1.81 | 1.22 | | 0.65 – 2.28 | 0.77 | 0.45 – 1.28 | 0.88 | 0.52 – 1.47 | 1.57 | 0.94 – 2.67 | 1.68 | 1.00 – 2.88 |
| Western Europe | 0.83 | 0.31 – 2.46 | 0.91 | 0.34 – 2.73 | 0.80 | 0.33 – 2.10 | 0.90 | | 0.36 – 2.42 | 1.07 | 0.47 – 2.56 | 1.16 | 0.51 – 2.81 | 1.04 | 0.42 – 2.40 | 1.08 | 0.43 – 2.51 |
| CCI (continuous) | - | - | 0.79 ** | 0.68 – 0.91 | - | - | 0.75 *** | | 0.66 – 0.86 | - | - | 0.80 *** | 0.72 – 0.89 | - | - | 0.88 * | 0.78 – 0.98 |
| CDI risk exposure  (future exposure) | 4.06 *** | 2.05 – 8.40 | 3.33 *** | 1.68 – 6.88 | 4.43 *** | 2.46 – 8.20 | 3.65 *** | | 2.02 – 6.76 | 1.22 | 0.77 – 1.93 | 1.04 | 0.65 – 1.65 | 1.12 | 0.71 – 1.75 | 1.03 | 0.65 – 1.62 |
| Baseline IgG  (above median) | 2.17 | 0.93 – 6.06 | 2.29 | 0.97 – 6.42 | 2.26 * | 1.06 – 5.44 | 2.45 * | | 1.14 – 5.94 | 1.63 | 0.96 – 2.83 | 1.70 | 1.01 – 2.98 | 2.09 ** | 1.30 – 3.33 | 2.14 ** | 1.34 – 3.43 |
| Vaccine group  (versus placebo) |  | |  | |  | | |  | |  |  |  |  |  |  |  |  |
| 100 µg + Al(OH)_3_ | 2492 *** | 506 –45418 | 2696 *** | 544 –49200 | 1651*** | 342 –29837 | 1897 *** | | 389 –34413 | § | § | § | § | § | § | § | § |
| 100 µg | 609 *** | 109 –11603 | 625  *** | 112 –11897 | 493 *** | 91 –9274 | 515 *** | | 95 –9701 | § | § | § | § | § | § | § | § |
| 50 µg + Al(OH)_3_ | 1820 *** | 285 –37324 | 1847 *** | 289 –37871 | 1285 *** | 218 –25178 | 1377 *** | | 232 –27056 | § | § | § | § | § | § | § | § |
| 50 µg | 565 *** | 102 –10738 | 580 *** | 105 –11031 | 417 *** | 78 –7815 | 444 *** | | 82 –8338 | § | § | § | § | § | § | § | § |

* p<0.05; ** p<0.01; *** p<0.001; § - Variable omitted due to complete separation (perfect prediction)

Al(OH)_3_ - Aluminium hydroxide; CCI - Charlson comorbidity index (modified); CDI - Clostridioides difficile infection; CI - Confidence interval; ELISA - Enzyme-linked immunosorbent assay; IgG - Immunoglobulin G; OR - Odds ratio; TNA - Toxin neutralization assay.

A hyphen (-) indicates that no association was found in any of the models.

Supplementary Table 5. Stepwise logistic regression multivariable model – ELISA and TNA measurements (Anti-toxin A IgG)

| Variable | Seroresponse - Toxin A (ELISA) | | | | | | | | | Seroresponse - Toxin A (TNA) | | | | | | | |
| --- | --- | --- | --- | --- | --- | --- | --- | --- | --- | --- | --- | --- | --- | --- | --- | --- | --- |
|  | Two-fold increase | | Four-fold increase | | Median fold increase | | | 75^th^ percentile fold increase | | Two-fold increase | | Four-fold increase | | Median fold increase | | 75^th^ percentile fold increase | |
|  | OR | CI | OR | CI | OR | CI | OR | | CI | OR | CI | OR | CI | OR | CI | OR | CI |
| Age  (>65 years) | 0.52 * | 0.27 – 0.99 | 0.52 * | 0.29 – 0.91 | 0.61 ** | 0.43 – 0.86 | - | | - | - | - | - | - | - | - | - | - |
| Sex  (female) | - | - | - | - | - | - | 1.31 | | 0.93 – 1.85 | - | - | 1.75 * | 1.11 – 2.79 | 1.64 ** | 1.16 – 2.33 | 1.31 | 0.92 – 1.86 |
| Study region  (versus others) |  | |  | |  | | |  | |  |  |  |  |  |  |  |  |
| North America | - | - | - | - | - | - | 1.45 | | 0.93 – 2.31 | - | - | - | - | - | - | 1.71 * | 1.07 – 2.77 |
| Western Europe | - | - | - | - | - | - | 2.31 * | | 1.08 – 4.96 | - | - | - | - | - | - | 1.09 | 0.44 – 2.51 |
| CCI (continuous) | - | - | - | - | - | - | 0.87 ** | | 0.78 – 0.96 | 0.80 ** | 0.69 – 0.92 | 0.76 *** | 0.66 – 0.86 | 0.80 *** | 0.72 – 0.89 | 0.88 * | 0.78 – 0.98 |
| CDI risk exposure  (future exposure) | 3.22 ** | 1.47 – 7.59 | 3.63 *** | 1.86 – 7.45 | - | - | - | | - | 3.90 *** | 2.07 – 7.73 | 3.93 *** | 2.27 – 7.00 | - | - | - | - |
| Baseline IgG  (above median) | - | - | - | - | - | - | - | | - | 2.48 | 1.06 – 6.93 | 2.51 * | 1.17 – 6.09 | 1.67 | 0.99 – 2.91 | 2.14 ** | 1.34 – 3.43 |
| Vaccine group  (versus placebo) |  | |  | |  | | |  | |  |  |  |  |  |  |  |  |
| 100 µg + Al(OH)_3_ | 859  *** | 332 –2785 | 2582 *** | 534 –46679 | 503 *** | 111 –  8889 | 108  *** | | 24 –1914 | 2605 *** | 531 –47359 | 1882 *** | 387 –34101 | § | § | § | § |
| 100 µg | 292  *** | 83 –1344 | 1464 *** | 235 –29678 | 293 *** | 58 –  5353 | 75  *** | | 15 –1387 | 688 *** | 124 –13060 | 540 *** | 100 –  10159 | § | § | § | § |
| 50 µg + Al(OH)_3_ | 343  *** | 98 –1579 | 1721 *** | 276 –34883 | 216 *** | 44 –  3925 | 79  *** | | 16 – 1453 | 1977 *** | 314 –40323 | 1449 *** | 245 –28414 | § | § | § | § |
| 50 µg | 408  *** | 107 –2209 | 2050 *** | 306 –43598 | 200 *** | 40 –3655 | 40  *** | | 7 – 755 | 634 *** | 115 –12004 | 466 *** | 87 –8738 | § | § | § | § |

* p<0.05; ** p<0.01; *** p<0.001; § - Variable omitted due to complete separation (perfect prediction)

Al(OH)_3_ - Aluminium hydroxide; CCI - Charlson comorbidity index (modified); CDI - Clostridioides difficile infection; CI - Confidence interval; ELISA - Enzyme-linked immunosorbent assay; IgG - Immunoglobulin G; OR - Odds ratio; TNA - Toxin neutralization assay.

A hyphen (-) indicates that no association was found in any of the models.

Supplementary Table 6.1 Mixed-effects multivariable model (full model) – ELISA and TNA measurements (Anti-toxin A IgG)

| Variable | Seroresponse - Toxin A (ELISA) | | | | | | | | | Seroresponse - Toxin A (TNA) | | | | | | | |
| --- | --- | --- | --- | --- | --- | --- | --- | --- | --- | --- | --- | --- | --- | --- | --- | --- | --- |
|  | Two-fold increase | | Four-fold increase | | Median fold increase | | | 75^th^ percentile fold increase | | Two-fold increase | | Four-fold increase | | Median fold increase | | 75^th^ percentile fold increase | |
|  | OR | CI | OR | CI | OR | CI | OR | | CI | OR | CI | OR | CI | OR | CI | OR | CI |
| Age  (>65 years) | 0.67 | 0.28 – 1.65 | 0.67 | 0.30 – 1.47 | 0.65 | 0.40 – 1.06 | 0.89 | | 0.55 – 1.46 | 0.81 | 0.40 – 1.62 | 1.15 | 0.62 – 2.13 | 1.06 | 0.65 – 1.72 | 1.14 | 0.69 – 1.90 |
| Sex  (female) | 1.01 | 0.53 – 1.96 | 1.22 | 0.68 – 2.20 | 1.24 | 0.87 – 1.76 | 1.32 | | 0.94 – 1.86 | 1.20 | 0.71 – 2.02 | 1.57 | 0.98 – 2.51 | 1.66 ** | 1.17 – 2.36 | 1.41 | 0.98 – 2.01 |
| Study region  (versus others) |  | |  | |  | | |  | |  |  |  |  |  |  |  |  |
| North America | 0.84 | 0.34 – 2.07 | 1.05 | 0.51 – 2.17 | 1.03 | 0.61 – 1.73 | 1.28 | | 0.77 – 2.13 | 1.19 | 0.58 – 2.43 | 0.95 | 0.51 – 1.76 | 0.88 | 0.52 – 1.50 | 2.17 ** | 1.26 – 3.76 |
| Western Europe | 3.89 | 0.61 – 24.66 | 2.81 | 0.62 – 12.74 | 2.05 | 0.79 – 5.27 | 2.18 * | | 1.01 – 4.71 | 0.98 | 0.35 – 2.73 | 1.01 | 0.40 – 2.58 | 1.16 | 0.49 – 2.72 | 0.99 | 0.41 – 2.38 |
| CCI (continuous) | 0.90 | 0.69 – 1.17 | 0.89 | 0.71 – 1.11 | 0.95 | 0.82 – 1.11 | 0.89 | | 0.77 – 1.04 | 0.82 | 0.67 – 1.00 | 0.74 *** | 0.62 – 0.88 | 0.79 ** | 0.68 – 0.92 | 0.85 * | 0.72 – 0.99 |
| CDI risk exposure  (future exposure) | 1.40 | 0.55 – 3.54 | 1.46 | 0.69 – 3.11 | 1.10 | 0.68 – 1.76 | 1.28 | | 0.81 – 2.00 | 2.26 * | 1.03 – 4.91 | 1.96 * | 1.03 – 3.71 | 1.02 | 0.64 – 1.65 | 1.48 | 0.88 – 2.51 |
| Baseline IgG  (above median) | 1.39 | 0.52 – 3.73 | 0.79 | 0.36 – 1.74 | 0.76 | 0.46 – 1.24 | 0.95 | | 0.58 – 1.56 | 2.25 | 0.89 – 5.69 | 2.43 * | 1.07 – 5.52 | 1.71 | 1.00 – 2.94 | 2.23 ** | 1.38 – 3.60 |
| Vaccine group  (versus placebo) |  | |  | |  | | |  | |  |  |  |  |  |  |  |  |
| 100 µg + Al(OH)_3_ | 1683 *** | 397 –7127 | 13877 *** | 433 –444709 | 533 *** | 74 –3869 | 107 *** | | 15 –775 | 3310 *** | 385 –  28491 | 3283 *** | 354 –  30427 | § | § | § | § |
| 100 µg | 117 *** | 26 –  530 | 472 *** | 47 –4698 | 303 *** | 39 –2382 | 71  *** | | 9 – 557 | 331 *** | 36 –3024 | 220 *** | 26 –  1887 | § | § | § | § |
| 50 µg + Al(OH)_3_ | 142 *** | 31 –648 | 548 *** | 55 –5457 | 220 *** | 28 –1714 | 74  *** | | 10 – 581 | 984 *** | 96 –  10118 | 574 *** | 62 –  5298 | § | § | § | § |
| 50 µg | 167 *** | 33 –843 | 660  *** | 62 –7032 | 205  *** | 26 –1610 | 38  *** | | 5 – 309 | 309 *** | 34 –  2816 | 187 *** | 22 –  1596 | § | § | § | § |

* p<0.05; ** p<0.01; *** p<0.001; § - Variable omitted due to complete separation (perfect prediction)

Al(OH)_3_ - Aluminium hydroxide; CCI - Charlson comorbidity index (modified); CDI - Clostridioides difficile infection; CI - Confidence interval; ELISA - Enzyme-linked immunosorbent assay; IgG - Immunoglobulin G; OR - Odds ratio; TNA - Toxin neutralization assay.

Supplementary Table 6.2 Mixed-effects multivariable model (alternatively including either age or comorbidity index as independent variable) – ELISA measurements (Anti-toxin A IgG)

| Variable | Seroresponse - Toxin A (ELISA) | | | | | | | | | | | | | | | | |
| --- | --- | --- | --- | --- | --- | --- | --- | --- | --- | --- | --- | --- | --- | --- | --- | --- | --- |
|  | Two-fold increase | | Two-fold increase | | Four-fold increase | | | Four-fold increase | | Median fold increase | | Median fold increase | | 75^th^ percentile fold increase | | 75^th^ percentile fold increase | |
|  | OR | CI | OR | CI | OR | CI | OR | | CI | OR | CI | OR | CI | OR | CI | OR | CI |
| Age  (>65 years) | 0.53 | 0.28 – 1.02 | - | - | 0.50  * | 0.28 – 0.90 | - | | - | 0.58 ** | 0.41 – 0.82 | - | - | 0.69 * | 0.49 – 0.97 | - | - |
| Sex  (female) | 1.04 | 0.54 – 2.00 | 0.99 | 0.52 – 1.91 | 1.25 | 0.69 – 2.25 | 1.20 | | 0.67 – 2.16 | 1.25 | 0.88 – 1.76 | 1.22 | 0.86 – 1.73 | 1.33 | 0.95 – 1.87 | 1.31 | 0.93 – 1.85 |
| Study region  (versus others) |  | |  | |  | | |  | |  |  |  |  |  |  |  |  |
| North America | 0.78 | 0.32 – 1.88 | 0.89 | 0.37 – 2.18 | 0.96 | 0.47 – 1.94 | 1.11 | | 0.54 – 2.28 | 0.99 | 0.60 – 1.66 | 1.09 | 0.65 – 1.83 | 1.20 | 0.73 – 1.99 | 1.30 | 0.79 – 2.16 |
| Western Europe | 3.67 | 0.59 – 22.79 | 3.99 | 0.62 – 25.50 | 2.67 | 0.59 – 12.00 | 2.84 | | 0.63 – 12.90 | 2.01 | 0.78 – 5.17 | 2.07 | 0.80 – 5.33 | 2.11 | 0.98 – 4.55 | 2.18 * | 1.01 – 4.71 |
| CCI (continuous) | - | - | 0.83 | 0.69 – 1.01 | - | - | 0.82  * | | 0.70 – 0.97 | - | - | 0.87 ** | 0.78 – 0.96 | - | - | 0.87 * | 0.78 – 0.97 |
| CDI risk exposure  (future exposure) | 1.52 | 0.62 – 3.75 | 1.29 | 0.52 – 3.21 | 1.61 | 0.77 – 3.36 | 1.35 | | 0.64 – 2.81 | 1.14 | 0.72 – 1.81 | 1.01 | 0.63 – 1.60 | 1.38 | 0.89 – 2.13 | 1.25 | 0.80 – 1.94 |
| Baseline IgG  (above median) | 1.40 | 0.53 – 3.73 | 1.42 | 0.53 – 3.81 | 0.81 | 0.37 – 1.76 | 0.80 | | 0.37 – 1.75 | 0.76 | 0.46 – 1.25 | 0.77 | 0.47 – 1.26 | 0.96 | 0.59 – 1.58 | 0.96 | 0.58 – 1.57 |
| Vaccine group  (versus placebo) |  | |  | |  | | |  | |  |  |  |  |  |  |  |  |
| 100 µg + Al(OH)_3_ | 1641 *** | 388 –6940 | 1692 *** | 401 –7147 | 13234 *** | 415 –  422121 | 13990 *** | | 437 –  448295 | 529 *** | 73 –  3838 | 542 *** | 75 –  3931 | 105 *** | 15 – 760 | 108 *** | 15 – 781 |
| 100 µg | 114 *** | 25 –516 | 118 *** | 26 –  534 | 454 *** | 46 –  4519 | 474 *** | | 48 –  4700 | 302  *** | 38 –  2381 | 308 *** | 39 –  2424 | 70 *** | 9 – 555 | 71 *** | 9 – 561 |
| 50 µg + Al(OH)_3_ | 140 *** | 31 –638 | 141 *** | 31 –  638 | 535 *** | 54 –  5328 | 538 *** | | 54 –  5339 | 221  *** | 28 –  1723 | 220 *** | 28 –  1714 | 75 *** | 10 – 585 | 75 *** | 10 – 583 |
| 50 µg | 163 *** | 33 –  821 | 167 *** | 33 –  834 | 637 *** | 60 –  6783 | 652 *** | | 62 –  6923 | 206 *** | 26 –  1616 | 207 *** | 26 –  1620 | 38 *** | 5 – 312 | 38 *** | 5 – 310 |

* p<0.05 ** p<0.01 *** p<0.001

Al(OH)_3_ - Aluminium hydroxide; CCI - Charlson comorbidity index (modified); CDI - Clostridioides difficile infection; CI - Confidence interval; ELISA - Enzyme-linked immunosorbent assay; IgG - Immunoglobulin G; OR - Odds ratio; TNA - Toxin neutralization assay.

A hyphen (-) indicates that no association was found in any of the models.

Supplementary Table 6.3 Mixed-effects multivariable model (alternatively including either age or comorbidity index as independent variable) – TNA measurements (Anti-toxin A IgG)

| Variable | Seroresponse - Toxin A (TNA) | | | | | | | | | | | | | | | | |
| --- | --- | --- | --- | --- | --- | --- | --- | --- | --- | --- | --- | --- | --- | --- | --- | --- | --- |
|  | Two-fold increase | | Two-fold increase | | Four-fold increase | | | Four-fold increase | | Median fold increase | | Median fold increase | | 75^th^ percentile fold increase | | 75^th^ percentile fold increase | |
|  | OR | CI | OR | CI | OR | CI | OR | | CI | OR | CI | OR | CI | OR | CI | OR | CI |
| Age  (>65 years) | 0.51 ** | 0.30 – 0.85 | - | - | 0.56 * | 0.36 – 0.88 | - | | - | 0.62 ** | 0.44 – 0.87 | - | - | 0.78 | 0.55 – 1.12 | - | - |
| Sex  (female) | 1.24 | 0.74 – 2.08 | 1.18 | 0.70 – 1.99 | 1.64 * | 1.03 – 2.60 | 1.58 | | 0.99 – 2.52 | 1.70 ** | 1.20 – 2.41 | 1.66 ** | 1.17 – 2.36 | 1.43 * | 1.00 – 2.04 | 1.41 | 0.99 – 2.02 |
| Study region  (versus others) |  | |  | |  | | |  | |  |  |  |  |  |  |  |  |
| North America | 1.03 | 0.51 – 2.05 | 1.23 | 0.61 – 2.49 | 0.76 | 0.42 – 1.39 | 0.93 | | 0.50 – 1.71 | 0.77 | 0.46 – 1.29 | 0.88 | 0.52 – 1.48 | 1.98 * | 1.16 – 3.37 | 2.13 ** | 1.25 – 3.65 |
| Western Europe | 0.90 | 0.33 – 2.49 | 0.98 | 0.35 – 2.74 | 0.90 | 0.36 – 2.25 | 1.01 | | 0.40 – 2.57 | 1.07 | 0.46 – 2.47 | 1.16 | 0.49 – 2.71 | 0.95 | 0.40 – 2.27 | 0.99 | 0.41 – 2.37 |
| CCI (continuous) | - | - | 0.79 ** | 0.68 – 0.91 | - | - | 0.76 *** | | 0.66 – 0.86 | - | - | 0.80 *** | 0.72 – 0.89 | - | - | 0.87 * | 0.78 – 0.98 |
| CDI risk exposure  (future exposure) | 2.60 * | 1.22 – 5.58 | 2.16 * | 1.00 – 4.66 | 2.43 ** | 1.31 – 4.52 | 2.01 * | | 1.07 – 3.77 | 1.22 | 0.77 – 1.93 | 1.04 | 0.65 – 1.65 | 1.65 * | 1.01 – 2.71 | 1.52 | 0.92 – 2.51 |
| Baseline IgG  (above median) | 2.16 | 0.86 – 5.44 | 2.26 | 0.89 – 5.73 | 2.25 * | 1.00 – 5.06 | 2.42 * | | 1.06 – 5.49 | 1.63 | 0.95 – 2.78 | 1.7 | 0.99 – 2.93 | 2.15 ** | 1.34 – 3.45 | 2.21 ** | 1.37 – 3.55 |
| Vaccine group  (versus placebo) |  | |  | |  | | |  | |  |  |  |  |  |  |  |  |
| 100 µg + Al(OH)_3_ | 3065 *** | 358 –  26212 | 3313 *** | 386 –  28467 | 2782 *** | 305 –  25411 | 3270 *** | | 353 –  30330 | § | § | § | § | § | § | § | § |
| 100 µg | 316 *** | 35 –  2889 | 334 *** | 37 –  3044 | 199 *** | 23 –  1709 | 218 *** | | 26 –  1873 | § | § | § | § | § | § | § | § |
| 50 µg + Al(OH)_3_ | 938 *** | 91 –  9643 | 977 *** | 95 –  10039 | 514 *** | 56 –  4726 | 575 *** | | 62 –  5312 | § | § | § | § | § | § | § | § |
| 50 µg | 292 *** | 32 –  2658 | 308 *** | 34 –  2806 | 168 *** | 20 –  1428 | 187 *** | | 22 –  1598 | § | § | § | § | § | § | § | § |

* p<0.05; ** p<0.01; *** p<0.001; § - Variable omitted due to complete separation (perfect prediction)

Al(OH)_3_ - Aluminium hydroxide; CCI - Charlson comorbidity index (modified); CDI - Clostridioides difficile infection; CI - Confidence interval; ELISA - Enzyme-linked immunosorbent assay; IgG - Immunoglobulin G; OR - Odds ratio; TNA - Toxin neutralization assay.

A hyphen (-) indicates that no association was found in any of the models.

Supplementary Table 7. Variable importance of classification and regression tree model – ELISA and TNA measurements (Anti-toxin A IgG)

| Variable | Seroresponse - Toxin A (ELISA) | | | | Seroresponse - Toxin A (TNA) | | | |
| --- | --- | --- | --- | --- | --- | --- | --- | --- |
|  | Two-fold increase | Four-fold increase | Median fold increase | 75^th^ percentile fold increase | Two-fold increase | Four-fold increase | Median fold increase | 75^th^ percentile fold increase |
| Age | 0 | 0 | 0 | § | 0 | 0 | 0 | 0 |
| Sex | 0 | 0 | 0 | § | 0 | 0 | 0 | 0 |
| Study region | 0 | 0 | 0 | § | 0 | 0 | 0 | 0.9 |
| CCI | 0 | 0 | 0 | § | 0 | 0 | 0 | 1.1 |
| CDI risk exposure | 0 | 0 | 0 | § | 0 | 0 | 0 | 2.8 |
| Baseline IgG | 0 | 0 | 0 | § | 0 | 0 | 0 | 6.4 |
| Vaccine group | 246.2 | 242.8 | 134.0 | § | 228.1 | 204.7 | 130.8 | 32.4 |

Variable importance is measured as decrease in impurity. § - Model convergence issues due to complete separation (perfect prediction).

CCI - Charlson comorbidity index (modified); CDI - Clostridioides difficile infection; ELISA - Enzyme-linked immunosorbent assay; IgG - Immunoglobulin G; TNA - Toxin neutralization assay.

Supplementary Table 8. Variable importance of boosting model – ELISA and TNA measurements (Anti-toxin A IgG)

| Variable | Seroresponse - Toxin A (ELISA) | | | | Seroresponse - Toxin A (TNA) | | | |
| --- | --- | --- | --- | --- | --- | --- | --- | --- |
|  | Two-fold increase | Four-fold increase | Median fold increase | 75^th^ percentile fold increase | Two-fold increase | Four-fold increase | Median fold increase | 75^th^ percentile fold increase |
| Age (>65 years) | 0.0000 | 0.0000 | 0.0045 | 0.0003 | 0.0000 | 0.0000 | 0.0000 | 0.0000 |
| Sex (female) | 0.0000 | 0.0000 | 0.0000 | 0.0014 | 0.0000 | 0.0007 | 0.0046 | 0.0013 |
| Study region |  |  |  |  |  |  |  |  |
| North America | 0.0000 | 0.0007 | 0.0000 | 0.0003 | 0.0044 | 0.0027 | 0.0000 | 0.0077 |
| Western Europe | 0.0000 | 0.0000 | 0.0000 | 0.0000 | 0.0000 | 0.0000 | 0.0000 | 0.0000 |
| CCI (continuous) | 0.0000 | 0.0000 | 0.0004 | 0.0034 | 0.0016 | 0.0064 | 0.0103 | 0.0031 |
| CDI risk exposure  (future exposure) | 0.0081 | 0.0154 | 0.0005 | 0.0038 | 0.0218 | 0.0302 | 0.0000 | 0.0004 |
| Baseline IgG  (above median) | 0.0000 | 0.0000 | 0.0000 | 0.0000 | 0.0000 | 0.0000 | 0.0011 | 0.0071 |
| Vaccine group |  |  |  |  |  |  |  |  |
| 100 µg + Al(OH)_3_ | 0.1984 | 0.1789 | 0.1304 | 0.0404 | 0.1733 | 0.1504 | 0.1246 | 0.0350 |
| 100 µg | 0.0405 | 0.0414 | 0.0211 | 0.0059 | 0.0331 | 0.0278 | 0.0178 | 0.0043 |
| 50 µg + Al(OH)_3_ | 0.0450 | 0.0464 | 0.0171 | 0.0067 | 0.0456 | 0.0399 | 0.0187 | 0.0036 |
| 50 µg | 0.0432 | 0.0440 | 0.0147 | 0.0011 | 0.0296 | 0.0239 | 0.0132 | 0.0033 |

Variable importance is measured as in-bag risk reduction.

Al(OH)_3_ - Aluminium hydroxide; CCI - Charlson comorbidity index (modified); CDI - Clostridioides difficile infection; ELISA - Enzyme-linked immunosorbent assay;

IgG - Immunoglobulin G; TNA - Toxin neutralization assay.

Supplementary Table 9.1 Logistic regression multivariable model (full model) – ELISA and TNA measurements (Anti-toxin B IgG)

| Variable | Seroresponse - Toxin B (ELISA) | | | | | | | | | Seroresponse - Toxin B (TNA) | | | | | | | |
| --- | --- | --- | --- | --- | --- | --- | --- | --- | --- | --- | --- | --- | --- | --- | --- | --- | --- |
|  | Two-fold increase | | Four-fold increase | | Median fold increase | | | 75^th^ percentile fold increase | | Two-fold increase | | Four-fold increase | | Median fold increase | | 75^th^ percentile fold increase | |
|  | OR | CI | OR | CI | OR | CI | OR | | CI | OR | CI | OR | CI | OR | CI | OR | CI |
| Age  (>65 years) | 0.58 | 0.30 – 1.09 | 0.95 | 0.54 – 1.66 | 1.48 | 0.92 – 2.41 | 1.25 | | 0.76 – 2.07 | 0.83 | 0.48 – 1.46 | 0.69 | 0.39 – 1.23 | 0.74 | 0.45 – 1.23 | 1.20 | 0.69 – 2.10 |
| Sex  (female) | 1.69 * | 1.04 – 2.76 | 1.74 * | 1.15 – 2.67 | 1.55 * | 1.10 – 2.19 | 1.04 | | 0.74 – 1.47 | 1.90 ** | 1.28 – 2.81 | 2.11 *** | 1.41 – 3.17 | 1.70 ** | 1.19 – 2.43 | 1.39 | 0.95 – 2.04 |
| Study region  (versus others) |  | |  | |  | | |  | |  |  |  |  |  |  |  |  |
| North America | 2.08 * | 1.12 – 3.90 | 2.15 ** | 1.25 – 3.72 | 1.09 | 0.65 – 1.82 | 2.01 * | | 1.19 – 3.47 | 5.39 *** | 2.83 – 10.83 | 5.28 *** | 2.62 – 11.43 | 2.66 *** | 1.57 – 4.61 | 2.67 ** | 1.45 – 5.05 |
| Western Europe | 1.59 | 0.63 – 4.30 | 2.74 * | 1.13 – 7.24 | 2.41 | 1.02 – 6.13 | 2.81 * | | 1.27 – 6.19 | 1.91 | 0.65 – 5.25 | 1.66 | 0.48 – 5.18 | 1.49 | 0.62 – 3.42 | 2.04 | 0.77 – 5.07 |
| CCI (continuous) | 0.78 ** | 0.65 – 0.93 | 0.78 ** | 0.66 – 0.92 | 0.78 ** | 0.68 – 0.91 | 0.84 * | | 0.71 – 0.98 | 0.91 | 0.76 – 1.08 | 0.96 | 0.80 – 1.15 | 0.94 | 0.80 – 1.09 | 0.89 | 0.75 – 1.06 |
| CDI risk exposure  (future exposure) | 1.13 | 0.64 – 2.01 | 1.34 | 0.80 – 2.24 | 0.46 ** | 0.29 – 0.73 | 0.91 | | 0.58 – 1.44 | 2.19 ** | 1.30 – 3.73 | 2.39 ** | 1.37 – 4.25 | 0.92 | 0.58 – 1.47 | 0.64 | 0.38 – 1.06 |
| Baseline IgG  (above median) | 1.86 * | 1.13 – 3.12 | 1.72 * | 1.12 – 2.68 | 1.64 ** | 1.16 – 2.33 | 1.11 | | 0.78 – 1.57 | 34.4 *** | 16.09 – 86.49 | 46.0 *** | 21.25 – 117.49 | 19.3 *** | 11.17 – 35.73 | 7.84 *** | 5.16 – 12.08 |
| Vaccine group  (versus placebo) |  | |  | |  | | |  | |  |  |  |  |  |  |  |  |
| 100 µg + Al(OH)_3_ | 167 *** | 83 –  371 | 233 *** | 92 –  789 | 109 *** | 47 –  317 | 101 *** | | 22 – 1781 | 234 *** | 76 –  903 | 304 *** | 90 –  1324 | 15  *** | 8 –  30 | 32  *** | 13 – 110 |
| 100 µg | 381 *** | 96 –  2614 | 639 *** | 157 –  3729 | 93  *** | 34 –  309 | 71  *** | | 14 – 1303 | 433 *** | 116 –  1946 | 501 *** | 127 –  2458 | 32  *** | 13 –  84 | 41  *** | 13 – 157 |
| 50 µg + Al(OH)_3_ | 210 *** | 69 –  773 | 296 *** | 90 –  1235 | 92  *** | 33 –  303 | 59  *** | | 11 – 1081 | 260 *** | 74 –  1105 | 349 *** | 91 –  1661 | 20  *** | 8 –  48 | 22  *** | 7 – 87 |
| 50 µg | 146 *** | 50 –  501 | 252 *** | 76 –  1057 | 119 *** | 42 –  401 | 70  *** | | 13 – 1287 | 140 *** | 39 –  597 | 190 *** | 49 –  917 | 11  *** | 5 –  28 | 18  *** | 6 – 73 |

* p<0.05; ** p<0.01; *** p<0.001

Al(OH)_3_ - Aluminium hydroxide; CCI - Charlson comorbidity index (modified); CDI - Clostridioides difficile infection; CI - Confidence interval; ELISA - Enzyme-linked immunosorbent assay; IgG - Immunoglobulin G; OR - Odds ratio; TNA - Toxin neutralization assay.

Supplementary Table 9.2 Logistic regression multivariable model (alternatively including either age or comorbidity index as independent variable) – ELISA measurements (Anti-toxin B IgG)

| Variable | Seroresponse - Toxin B (ELISA) | | | | | | | | | | | | | | | | |
| --- | --- | --- | --- | --- | --- | --- | --- | --- | --- | --- | --- | --- | --- | --- | --- | --- | --- |
|  | Two-fold increase | | Two-fold increase | | Four-fold increase | | | Four-fold increase | | Median fold increase | | Median fold increase | | 75^th^ percentile fold increase | | 75^th^ percentile fold increase | |
|  | OR | CI | OR | CI | OR | CI | OR | | CI | OR | CI | OR | CI | OR | CI | OR | CI |
| Age  (>65 years) | 0.32 *** | 0.19 – 0.52 | - | - | 0.53 ** | 0.35 – 0.80 | - | | - | 0.84 | 0.60 – 1.18 | - | - | 0.83 | 0.59 – 1.17 | - | - |
| Sex  (female) | 1.74 * | 1.08 – 2.83 | 1.65 * | 1.02 – 2.69 | 1.78 ** | 1.18 – 2.72 | 1.74 * | | 1.14 – 2.66 | 1.58 ** | 1.13 – 2.23 | 1.57 ** | 1.12 – 2.21 | 1.06 | 0.75 – 1.49 | 1.05 | 0.74 – 1.48 |
| Study region  (versus others) |  | |  | |  | | |  | |  |  |  |  |  |  |  |  |
| North America | 1.75 | 0.95 – 3.21 | 2.26 ** | 1.22 – 4.20 | 1.81 * | 1.06 – 3.07 | 2.17 ** | | 1.26 – 3.73 | 0.95 | 0.57 – 1.56 | 1.03 | 0.62 – 1.70 | 1.82 * | 1.09 – 3.09 | 1.95 * | 1.16 – 3.34 |
| Western Europe | 1.42 | 0.58 – 3.75 | 1.63 | 0.65 – 4.43 | 2.47 | 1.03 – 6.43 | 2.74 * | | 1.14 – 7.26 | 2.20 | 0.94 – 5.53 | 2.37 | 1.01 – 5.98 | 2.66 * | 1.21 – 5.81 | 2.80 * | 1.27 – 6.16 |
| CCI (continuous) | - | - | 0.70 *** | 0.61 – 0.80 | - | - | 0.77 *** | | 0.68 – 0.87 | - | - | 0.85 ** | 0.77 – 0.95 | - | - | 0.88 * | 0.79 – 0.98 |
| CDI risk exposure  (future exposure) | 1.36 | 0.78 – 2.38 | 1.02 | 0.58 – 1.78 | 1.60 | 0.97 – 2.63 | 1.33 | | 0.80 – 2.19 | 0.55 * | 0.35 – 0.87 | 0.50 ** | 0.31 – 0.79 | 1.03 | 0.66 – 1.60 | 0.95 | 0.61 – 1.49 |
| Baseline IgG  (above median) | 1.82 * | 1.11 – 3.06 | 1.90 * | 1.16 – 3.20 | 1.69 * | 1.10 – 2.62 | 1.73 * | | 1.12 – 2.69 | 1.63 ** | 1.16 – 2.31 | 1.62 ** | 1.14 – 2.29 | 1.11 | 0.78 – 1.57 | 1.11 | 0.78 – 1.57 |
| Vaccine group  (versus placebo) |  | |  | |  | | |  | |  |  |  |  |  |  |  |  |
| 100 µg + Al(OH)_3_ | 148 *** | 75 –  322 | 166 *** | 83 –  368 | 209 *** | 84 –  702 | 234 *** | | 93 –  791 | 101 *** | 44 –  292 | 105 *** | 46 –  304 | 97  *** | 21 – 1709 | 99  *** | 22 – 1747 |
| 100 µg | 360 *** | 91 –  2462 | 377 *** | 95 –  2576 | 605 *** | 149 –  3519 | 641 *** | | 158 –  3739 | 89  *** | 32 –  296 | 89  *** | 32 –  296 | 69  *** | 13 – 1279 | 69  *** | 13 – 1276 |
| 50 µg + Al(OH)_3_ | 200 *** | 66 –  729 | 203 *** | 67 –  740 | 282 *** | 86 –  1172 | 296 *** | | 90 –  1234 | 89  *** | 33 –  294 | 90  *** | 33 –  295 | 58  *** | 11 – 1075 | 58  *** | 11 – 1070 |
| 50 µg | 135 *** | 47 –  459 | 143 *** | 49 –  486 | 236 *** | 72 –  987 | 252 *** | | 76 –  1058 | 115 *** | 41 –  387 | 115 *** | 41 –390 | 69  *** | 13 – 1283 | 69  *** | 13 – 1271 |

* p<0.05; ** p<0.01; *** p<0.001

Al(OH)_3_ - Aluminium hydroxide; CCI - Charlson comorbidity index (modified); CDI - Clostridioides difficile infection; CI - Confidence interval; ELISA - Enzyme-linked immunosorbent assay; IgG - Immunoglobulin G; OR - Odds ratio; TNA - Toxin neutralization assay.

A hyphen (-) indicates that no association was found in any of the models.

Supplementary Table 9.3 Logistic regression multivariable model (alternatively including either age or comorbidity index as independent variable) – TNA measurements (Anti-toxin B IgG)

| Variable | Seroresponse - Toxin B (TNA) | | | | | | | | | | | | | | | |
| --- | --- | --- | --- | --- | --- | --- | --- | --- | --- | --- | --- | --- | --- | --- | --- | --- |
|  | Two-fold increase | | Two-fold increase | | Four-fold increase | | Four-fold increase | | Median fold increase | | Median fold increase | | 75^th^ percentile fold increase | | 75^th^ percentile fold increase | |
|  | OR | CI | OR | CI | OR | CI | OR | CI | OR | CI | OR | CI | OR | CI | OR | CI |
| Age  (>65 years) | 0.67  * | 0.45 – 1.00 | - | - | 0.63  * | 0.42 – 0.94 | - | - | 0.64  * | 0.44 – 0.91 | - | - | 0.92 | 0.63 – 1.35 | - | - |
| Sex  (female) | 1.91  ** | 1.30 – 2.84 | 1.88  ** | 1.27 – 2.79 | 2.12 *** | 1.42 – 3.19 | 2.07 *** | 1.39 – 3.11 | 1.71  ** | 1.20 – 2.44 | 1.68  ** | 1.18 – 2.40 | 1.40 | 0.95 – 2.05 | 1.40 | 0.95 – 2.05 |
| Study region  (versus others) |  | |  | |  | |  | |  |  |  |  |  |  |  |  |
| North America | 5.13 *** | 2.71 – 10.21 | 5.54 *** | 2.92 – 11.08 | 5.17 *** | 2.58 – 11.11 | 5.56 *** | 2.76 – 12.01 | 2.58 *** | 1.53 – 4.44 | 2.77 *** | 1.64 – 4.78 | 2.49 ** | 1.37 – 4.64 | 2.59 ** | 1.42 – 4.87 |
| Western Europe | 1.87 | 0.64 – 5.16 | 1.91 | 0.65 – 5.24 | 1.64 | 0.47 – 5.13 | 1.66 | 0.48 – 5.16 | 1.48 | 0.62 – 3.39 | 1.48 | 0.62 – 3.39 | 1.95 | 0.74 – 4.88 | 2.02 | 0.77 – 5.05 |
| CCI (continuous) | - | - | 0.88  * | 0.77 – 0.99 | - | - | 0.88 | 0.78 – 1.00 | - | - | 0.88  * | 0.79 – 0.98 | - | - | 0.93 | 0.83 – 1.05 |
| CDI risk exposure  (future exposure) | 2.31  ** | 1.39 – 3.90 | 2.12  ** | 1.27 – 3.58 | 2.45  ** | 1.42 – 4.31 | 2.24  ** | 1.30 – 3.95 | 0.96 | 0.61 – 1.51 | 0.87 | 0.55 – 1.37 | 0.69 | 0.42 – 1.14 | 0.66 | 0.40 – 1.09 |
| Baseline IgG  (above median) | 34.15 *** | 15.97 – 85.84 | 34.41 *** | 16.10 – 86.39 | 45.94 *** | 21.20 – 117.24 | 45.75 *** | 21.15 – 116.44 | 19.25 *** | 11.14 – 35.61 | 19.36 *** | 11.21 – 35.82 | 7.79 *** | 5.13 – 11.98 | 7.82 *** | 5.15 – 12.04 |
| Vaccine group  (versus placebo) |  | |  | |  | |  | |  |  |  |  |  |  |  |  |
| 100 µg + Al(OH)_3_ | 227 *** | 74 –  875 | 235 *** | 76 –  905 | 301 *** | 89 –  1308 | 305 *** | 91 –  1322 | 15  *** | 8 –  30 | 15  *** | 8 –  31 | 31  *** | 12 – 107 | 32  *** | 13 – 109 |
| 100 µg | 427 *** | 115 –  1916 | 438 *** | 118 –  1964 | 499 *** | 126 –  2448 | 506 *** | 128 –  2479 | 32  *** | 13 –  84 | 32  *** | 13 –  86 | 41  *** | 13 – 155 | 41  *** | 13 – 155 |
| 50 µg + Al(OH)_3_ | 259 *** | 73 –  1101 | 258 *** | 73 –  1092 | 349 *** | 91 –  1662 | 339 *** | 89 –  1607 | 20  *** | 8 –  48 | 19  *** | 8 –  48 | 22  *** | 7 – 86 | 22  *** | 7 – 86 |
| 50 µg | 138 *** | 39 –  589 | 139 *** | 39 –  594 | 190 *** | 49 –  914 | 188 *** | 48 –  901 | 11  *** | 5 –  28 | 11  *** | 5 –  28 | 19  *** | 6 – 73 | 18  *** | 6 – 73 |

* p<0.05; ** p<0.01; *** p<0.001

Al(OH)_3_ - Aluminium hydroxide; CCI - Charlson comorbidity index (modified); CDI - Clostridioides difficile infection; CI - Confidence interval; ELISA - Enzyme-linked immunosorbent assay; IgG - Immunoglobulin G; OR - Odds ratio; TNA - Toxin neutralization assay.

A hyphen (-) indicates that no association was found in any of the models.

Supplementary Table 10. Stepwise logistic regression multivariable model – ELISA and TNA measurements (Anti-toxin B IgG)

| Variable | Seroresponse - Toxin B (ELISA) | | | | | | | | | Seroresponse - Toxin B (TNA) | | | | | | | |
| --- | --- | --- | --- | --- | --- | --- | --- | --- | --- | --- | --- | --- | --- | --- | --- | --- | --- |
|  | Two-fold increase | | Four-fold increase | | Median fold increase | | | 75^th^ percentile fold increase | | Two-fold increase | | Four-fold increase | | Median fold increase | | 75^th^ percentile fold increase | |
|  | OR | CI | OR | CI | OR | CI | OR | | CI | OR | CI | OR | CI | OR | CI | OR | CI |
| Age  (>65 years) | 0.59 | 0.31 – 1.11 | - | - | 1.48 | 0.92 – 2.41 | - | | - | - | - | 0.63  * | 0.42 – 0.94 | 0.64  * | 0.44 – 0.91 | - | - |
| Sex  (female) | 1.68 * | 1.04 – 2.75 | 1.74 * | 1.14 – 2.66 | 1.55 * | 1.10 – 2.19 | - | | - | 1.88  ** | 1.27 – 2.79 | 2.12 *** | 1.42 – 3.19 | 1.71  ** | 1.20 – 2.44 | 1.40 | 0.95 – 2.05 |
| Study region  (versus others) |  | |  | |  | | |  | |  |  |  |  |  |  |  |  |
| North America | 2.20 ** | 1.25 – 3.91 | 2.47 *** | 1.51 – 4.04 | 1.09 | 0.65 – 1.82 | 1.92 ** | | 1.21 – 3.13 | 5.54 *** | 2.92 – 11.08 | 5.17 *** | 2.58 – 11.11 | 2.54 *** | 1.58 – 4.15 | 2.49 ** | 1.38 – 4.65 |
| Western Europe | 1.64 | 0.66 – 4.40 | 2.94 * | 1.22 – 7.74 | 2.41 | 1.02 – 6.13 | 2.71 * | | 1.24 – 5.90 | 1.91 | 0.65 – 5.24 | 1.64 | 0.47 – 5.13 | 1.47 | 0.61 – 3.34 | 1.94 | 0.73 – 4.82 |
| CCI (continuous) | 0.77 ** | 0.64 – 0.92 | 0.77 *** | 0.68 – 0.86 | 0.78 ** | 0.68 – 0.91 | 0.88 * | | 0.79 – 0.98 | 0.88  * | 0.77 – 0.99 | - | - | - | - | - | - |
| CDI risk exposure  (future exposure) | - | - | - | - | 0.46 ** | 0.29 – 0.73 | - | | - | 2.12  ** | 1.27 – 3.58 | 2.45  ** | 1.42 – 4.31 | - | - | 0.69 | 0.42 – 1.13 |
| Baseline IgG  (above median) | 1.86 * | 1.13 – 3.14 | 1.73 * | 1.13 – 2.70 | 1.64 ** | 1.16 – 2.33 | - | | - | 34.41 *** | 16.10 – 86.39 | 45.94 *** | 21.20 – 117.24 | 19.23 *** | 11.13 – 35.55 | 7.79 *** | 5.13 – 11.99 |
| Vaccine group  (versus placebo) |  | |  | |  | | |  | |  |  |  |  |  |  |  |  |
| 100 µg + Al(OH)_3_ | 168 *** | 83 –  373 | 234 *** | 93 –  790 | 109 *** | 47 –  317 | 98  *** | | 22 – 1738 | 235 *** | 76 –  906 | 301 *** | 89 –  1308 | 15  *** | 8 –  30 | 31  *** | 12 – 107 |
| 100 µg | 396 *** | 101 –  2700 | 695 *** | 172 –  4036 | 93  *** | 34 –  309 | 69  *** | | 13 – 1260 | 438 *** | 118 –  1964 | 499 *** | 126 –  2448 | 31  *** | 13 –  82 | 41  *** | 13 – 156 |
| 50 µg + Al(OH)_3_ | 219 *** | 73 –  795 | 321 *** | 99 –  1334 | 92  *** | 33 –  303 | 56  *** | | 11 –1032 | 258 *** | 73 –  1092 | 349 *** | 91 –  1662 | 19  *** | 8 –  47 | 22  *** | 7 – 86 |
| 50 µg | 152 *** | 52 –  515 | 274 *** | 84 –  1144 | 119 *** | 42 –  401 | 66  *** | | 13 – 1221 | 139 *** | 39 –  594 | 190 *** | 49 –  914 | 11  *** | 5 –  27 | 19  *** | 6 – 73 |

* p<0.05; ** p<0.01; *** p<0.001

Al(OH)_3_ - Aluminium hydroxide; CCI - Charlson comorbidity index (modified); CDI - Clostridioides difficile infection; CI - Confidence interval; ELISA - Enzyme-linked immunosorbent assay; IgG - Immunoglobulin G; OR - Odds ratio; TNA - Toxin neutralization assay.

A hyphen (-) indicates that no association was found in any of the models.

Supplementary Table 11.1 Mixed-effects multivariable model (full model) – ELISA and TNA measurements (Anti-toxin B IgG)

| Variable | Seroresponse - Toxin B (ELISA) | | | | | | | | | Seroresponse - Toxin B (TNA) | | | | | | | |
| --- | --- | --- | --- | --- | --- | --- | --- | --- | --- | --- | --- | --- | --- | --- | --- | --- | --- |
|  | Two-fold increase | | Four-fold increase | | Median fold increase | | | 75^th^ percentile fold increase | | Two-fold increase | | Four-fold increase | | Median fold increase | | 75^th^ percentile fold increase | |
|  | OR | CI | OR | CI | OR | CI | OR | | CI | OR | CI | OR | CI | OR | CI | OR | CI |
| Age  (>65 years) | 0.57 | 0.30 – 1.10 | 0.95 | 0.54 – 1.68 | 1.49 | 0.92 – 2.41 | 1.24 | | 0.75 – 2.06 | 0.82 | 0.46 – 1.48 | 0.67 | 0.37 – 1.23 | 0.74 | 0.45 – 1.23 | 1.20 | 0.69 – 2.09 |
| Sex  (female) | 1.54 | 0.94 – 2.51 | 1.56  * | 1.01 – 2.40 | 1.68 ** | 1.18 – 2.39 | 1.07 | | 0.75 – 1.52 | 1.71  ** | 1.14 – 2.56 | 1.92  ** | 1.26 – 2.92 | 1.70  ** | 1.19 – 2.42 | 1.44 | 0.98 – 2.13 |
| Study region  (versus others) |  | |  | |  | | |  | |  |  |  |  |  |  |  |  |
| North America | 1.56 | 0.82 – 2.96 | 1.54 | 0.88 – 2.68 | 1.44 | 0.82 – 2.51 | 2.19 ** | | 1.24 – 3.88 | 3.56 *** | 1.75 – 7.26 | 3.47  ** | 1.59 – 7.60 | 2.66 *** | 1.55 – 4.56 | 2.99 *** | 1.58 – 5.68 |
| Western Europe | 1.76 | 0.67 – 4.60 | 3.11  * | 1.22 – 7.89 | 2.22 | 0.91 – 5.43 | 2.73 * | | 1.24 – 6.02 | 2.27 | 0.81 – 6.35 | 2.01 | 0.62 – 6.49 | 1.49 | 0.64 – 3.48 | 1.94 | 0.76 – 4.96 |
| CCI (continuous) | 0.78 ** | 0.65 – 0.94 | 0.78  ** | 0.66 – 0.92 | 0.78 *** | 0.67 – 0.90 | 0.84 * | | 0.72 – 0.98 | 0.91 | 0.76 – 1.09 | 0.96 | 0.80 – 1.16 | 0.94 | 0.81 – 1.09 | 0.89 | 0.75 – 1.06 |
| CDI risk exposure  (future exposure) | 0.77 | 0.41 – 1.43 | 0.82 | 0.47 – 1.42 | 0.67 | 0.39 – 1.16 | 1.05 | | 0.59 – 1.87 | 1.02 | 0.54 – 1.92 | 1.07 | 0.53 – 2.15 | 0.92 | 0.58 – 1.47 | 0.81 | 0.43 – 1.50 |
| Baseline IgG  (above median) | 1.96 * | 1.17 – 3.27 | 1.83  ** | 1.18 – 2.86 | 1.62 ** | 1.14 – 2.30 | 1.10 | | 0.78 – 1.56 | 36.50 *** | 15.95 – 83.53 | 49.27 *** | 21.24 – 114.28 | 19.31 *** | 10.84 – 34.41 | 8.09 *** | 5.26 – 12.44 |
| Vaccine group  (versus placebo) |  | |  | |  | | |  | |  |  |  |  |  |  |  |  |
| 100 µg + Al(OH)_3_ | 173 *** | 81 –  373 | 258  *** | 88 –  757 | 123 *** | 48 –  315 | 105 *** | | 15 – 760 | 228 *** | 65 –  798 | 290 *** | 75 –  1115 | 15  *** | 8 –  29 | 36  *** | 12 – 104 |
| 100 µg | 214 *** | 42 –  1097 | 319  *** | 65 –  1569 | 147 *** | 47 –  461 | 84  *** | | 10 – 693 | 219 *** | 52 –  914 | 263 *** | 59 –  1174 | 32  *** | 12 –  81 | 55  *** | 15 – 204 |
| 50 µg + Al(OH)_3_ | 117 *** | 33 –  416 | 147  *** | 39 –  559 | 146 *** | 47 –  454 | 70  *** | | 8 – 574 | 130 *** | 33 –  515 | 182 *** | 42 –  788 | 20  *** | 8 –  47 | 30  *** | 8 – 114 |
| 50 µg | 82  *** | 24 –  278 | 127  *** | 33 –  485 | 188 *** | 59 –  600 | 83  *** | | 10 – 684 | 70  *** | 18 –  281 | 100 *** | 23 –  439 | 11  *** | 4 –  27 | 25  *** | 6 – 95 |

* p<0.05; ** p<0.01; *** p<0.001

Al(OH)_3_ - Aluminium hydroxide; CCI - Charlson comorbidity index (modified); CDI - Clostridioides difficile infection; CI - Confidence interval; ELISA - Enzyme-linked immunosorbent assay; IgG - Immunoglobulin G; OR - Odds ratio; TNA - Toxin neutralization assay.

Supplementary Table 11.2 Mixed-effects multivariable model (alternatively including either age or comorbidity index as independent variable) – ELISA measurements (Anti-toxin B IgG)

| Variable | Seroresponse - Toxin B (ELISA) | | | | | | | | | | | | | | | | |
| --- | --- | --- | --- | --- | --- | --- | --- | --- | --- | --- | --- | --- | --- | --- | --- | --- | --- |
|  | Two-fold increase | | Two-fold increase | | Four-fold increase | | | Four-fold increase | | Median fold increase | | Median fold increase | | 75^th^ percentile fold increase | | 75^th^ percentile fold increase | |
|  | OR | CI | OR | CI | OR | CI | OR | | CI | OR | CI | OR | CI | OR | CI | OR | CI |
| Age  (>65 years) | 0.32 *** | 0.20 – 0.53 | - | - | 0.53  ** | 0.35 – 0.81 | - | | - | 0.83 | 0.59 – 1.17 | - | - | 0.83 | 0.59 – 1.17 | - | - |
| Sex  (female) | 1.59 | 0.98 – 2.59 | 1.50 | 0.92 – 2.45 | 1.60  * | 1.05 – 2.46 | 1.56 * | | 1.01 – 2.39 | 1.71 ** | 1.21 – 2.42 | 1.70 ** | 1.20 – 2.41 | 1.08 | 0.76 – 1.54 | 1.07 | 0.76 – 1.53 |
| Study region  (versus others) |  | |  | |  | | |  | |  |  |  |  |  |  |  |  |
| North America | 1.31 | 0.70 – 2.44 | 1.69 | 0.90 – 3.19 | 1.30 | 0.76 – 2.23 | 1.55 | | 0.90 – 2.69 | 1.23 | 0.71 – 2.12 | 1.35 | 0.78 – 2.35 | 1.97 * | 1.12 – 3.46 | 2.12 ** | 1.21 – 3.74 |
| Western Europe | 1.57 | 0.61 – 4.04 | 1.80 | 0.68 – 4.73 | 2.80  * | 1.12 – 7.00 | 3.11 * | | 1.23 – 7.91 | 2.03 | 0.84 – 4.91 | 2.18 | 0.89 – 5.30 | 2.58 * | 1.18 – 5.67 | 2.72 * | 1.23 – 5.99 |
| CCI (continuous) | - | - | 0.70 *** | 0.61 – 0.81 | - | - | 0.77 *** | | 0.68 – 0.87 | - | - | 0.85 ** | 0.76 – 0.94 | - | - | 0.88 * | 0.79 – 0.98 |
| CDI risk exposure  (future exposure) | 0.92 | 0.50 – 1.69 | 0.69 | 0.37 – 1.27 | 0.98 | 0.58 – 1.67 | 0.81 | | 0.47 – 1.39 | 0.8 | 0.47 – 1.36 | 0.72 | 0.42 – 1.24 | 1.18 | 0.67 – 2.08 | 1.10 | 0.62 – 1.93 |
| Baseline IgG  (above median) | 1.93 * | 1.16 – 3.21 | 2.00 ** | 1.20 – 3.34 | 1.79  ** | 1.15 – 2.77 | 1.84 ** | | 1.18 – 2.86 | 1.61 ** | 1.14 – 2.29 | 1.60 ** | 1.12 – 2.27 | 1.10 | 0.78 – 1.56 | 1.10 | 0.77 – 1.55 |
| Vaccine group  (versus placebo) |  | |  | |  | | |  | |  |  |  |  |  |  |  |  |
| 100 µg + Al(OH)_3_ | 153 *** | 73 –  323 | 173  *** | 81 –  370 | 228  *** | 79 –660 | 259  *** | | 88 –  758 | 113  *** | 44 –  287 | 118 *** | 46 –  302 | 101  *** | 14 – 727 | 103 *** | 14 – 745 |
| 100 µg | 199 *** | 39 –1017 | 211  *** | 41 –  1078 | 297  *** | 61 –1458 | 320  *** | | 65 –  1572 | 139  *** | 45 –  432 | 141  *** | 45 –  440 | 82  *** | 10 – 675 | 82  *** | 10 – 679 |
| 50 µg + Al(OH)_3_ | 110 *** | 31 –  387 | 113  *** | 32 –  398 | 138  *** | 37 –  522 | 147  *** | | 39 –559 | 140  *** | 45 –  433 | 142 *** | 46 –  442 | 69  *** | 8 – 568 | 69  *** | 8 – 569 |
| 50 µg | 75  *** | 22 –251 | 80  *** | 24 –  270 | 117  *** | 31 –  444 | 127  *** | | 33 –  485 | 179  *** | 56 –  567 | 182 *** | 57 –  581 | 82  *** | 10 – 677 | 82  *** | 10 – 676 |

* p<0.05; ** p<0.01; *** p<0.001

Al(OH)_3_ - Aluminium hydroxide; CCI - Charlson comorbidity index (modified); CDI - Clostridioides difficile infection; CI - Confidence interval; ELISA - Enzyme-linked immunosorbent assay; IgG - Immunoglobulin G; OR - Odds ratio; TNA - Toxin neutralization assay.

A hyphen (-) indicates that no association was found in any of the models.

Supplementary Table 11.3 Mixed-effects multivariable model (alternatively including either age or comorbidity index as independent variable) – TNA measurements (Anti-toxin B IgG)

| Variable | Seroresponse - Toxin B (TNA) | | | | | | | | | | | | | | | | |
| --- | --- | --- | --- | --- | --- | --- | --- | --- | --- | --- | --- | --- | --- | --- | --- | --- | --- |
|  | Two-fold increase | | Two-fold increase | | Four-fold increase | | | Four-fold increase | | Median fold increase | | Median fold increase | | 75^th^ percentile fold increase | | 75^th^ percentile fold increase | |
|  | OR | CI | OR | CI | OR | CI | OR | | CI | OR | CI | OR | CI | OR | CI | OR | CI |
| Age  (>65 years) | 0.66 | 0.44 – 1.00 | - | - | 0.61  * | 0.40 – 0.94 | - | | - | 0.64  * | 0.45 – 0.91 | - | - | 0.92 | 0.63 – 1.35 | - | - |
| Sex  (female) | 1.73  ** | 1.15 – 2.59 | 1.69  * | 1.13 – 2.54 | 1.93  ** | 1.27 – 2.93 | 1.88  ** | | 1.24 – 2.85 | 1.71  ** | 1.20 – 2.44 | 1.68  ** | 1.18 – 2.39 | 1.45 | 0.99 – 2.14 | 1.45 | 0.98 – 2.14 |
| Study region  (versus others) |  | |  | |  | | |  | |  |  |  |  |  |  |  |  |
| North America | 3.38 *** | 1.68 – 6.82 | 3.66 *** | 1.81 – 7.43 | 3.40  ** | 1.56 – 7.38 | 3.67  ** | | 1.68 – 8.01 | 2.58 *** | 1.52 – 4.40 | 2.77 *** | 1.62 – 4.73 | 2.79 ** | 1.49 – 5.23 | 2.91 *** | 1.54 – 5.48 |
| Western Europe | 2.23 | 0.79 – 6.25 | 2.27 | 0.81 – 6.33 | 1.99 | 0.61 – 6.45 | 2.01 | | 0.63 – 6.46 | 1.48 | 0.63 – 3.46 | 1.48 | 0.64 – 3.45 | 1.86 | 0.73 – 4.77 | 1.93 | 0.75 – 4.94 |
| CCI (continuous) | - | - | 0.87  * | 0.77 – 0.99 | - | - | 0.88 | | 0.77 – 1.00 | - | - | 0.88  * | 0.79 – 0.98 | - | - | 0.93 | 0.83 – 1.05 |
| CDI risk exposure  (future exposure) | 1.08 | 0.58 – 2.02 | 0.98 | 0.53 – 1.85 | 1.10 | 0.55 – 2.18 | 1.00 | | 0.50 – 1.99 | 0.96 | 0.61 – 1.51 | 0.87 | 0.56 – 1.37 | 0.88 | 0.48 – 1.60 | 0.84 | 0.45 – 1.54 |
| Baseline IgG  (above median) | 36.27 *** | 15.85 – 82.98 | 36.52 *** | 15.97 – 83.51 | 49.20 *** | 21.21 – 114.13 | 48.98 *** | | 21.16 – 113.36 | 19.25 *** | 10.81 – 34.30 | 19.36 *** | 10.87 – 34.50 | 8.03 *** | 5.22 – 12.34 | 8.06 *** | 5.24 – 12.40 |
| Vaccine group  (versus placebo) |  | |  | |  | | |  | |  |  |  |  |  |  |  |  |
| 100 µg + Al(OH)_3_ | 220 *** | 63 –  765 | 230 *** | 66 –  804 | 286  *** | 75 –  1097 | 292 *** | | 76 –  1123 | 15  *** | 8 –  29 | 15  *** | 8 –  29 | 35  *** | 12 – 102 | 35  *** | 12 – 103 |
| 100 µg | 213 *** | 51 –  891 | 222 *** | 53 –  926 | 261  *** | 59 –  1164 | 268 *** | | 60 –  1195 | 32  *** | 12 –  81 | 32  *** | 13 –  83 | 55  *** | 15 – 202 | 55  *** | 15 – 202 |
| 50 µg + Al(OH)_3_ | 129 *** | 33 –  508 | 129 *** | 33 –  511 | 182  *** | 42 –  785 | 179 *** | | 42 –  768 | 20  *** | 8 –  47 | 19  *** | 8 –  46 | 30  *** | 8 –113 | 30  *** | 8 – 114 |
| 50 µg | 69  *** | 17 –  275 | 70  *** | 18 –281 | 99  *** | 23 –  436 | 100 *** | | 23 –  436 | 11  *** | 4 –  27 | 11  *** | 5 –  27 | 25  *** | 6 – 96 | 25  *** | 7 – 95 |

* p<0.05; ** p<0.01; *** p<0.001

Al(OH)_3_ - Aluminium hydroxide; CCI - Charlson comorbidity index (modified); CDI - Clostridioides difficile infection; CI - Confidence interval; ELISA - Enzyme-linked immunosorbent assay; IgG - Immunoglobulin G; OR - Odds ratio; TNA - Toxin neutralization assay.

A hyphen (-) indicates that no association was found in any of the models.

Supplementary Table 12. Variable importance of classification and regression tree model – ELISA and TNA measurements (Anti-toxin B IgG)

| Variable | Seroresponse - Toxin B (ELISA) | | | | Seroresponse - Toxin B (TNA) | | | |
| --- | --- | --- | --- | --- | --- | --- | --- | --- |
|  | Two-fold increase | Four-fold increase | Median fold increase | 75^th^ percentile fold increase | Two-fold increase | Four-fold increase | Median fold increase | 75^th^ percentile fold increase |
| Age | 0 | 0 | 0 | § | 0 | 1.9 | 2.8 | 0 |
| Sex | 0 | 0 | 0 | § | 3.3 | 7.0 | 3.0 | 0 |
| Study region | 0 | 0 | 0 | § | 29.2 | 25.6 | 11.3 | 0 |
| CCI | 0 | 0 | 5.8 | § | 0.4 | 1.5 | 1.8 | 0 |
| CDI risk exposure | 0 | 0 | 0 | § | 17.9 | 7.8 | 3.4 | 0 |
| Baseline IgG | 0 | 0 | 0 | § | 79.6 | 76.8 | 83.5 | 44.3 |
| Vaccine group | 199.0 | 182.2 | 122.4 | § | 70.7 | 71.7 | 44.5 | 27.8 |

Variable importance is measured as decrease in impurity. § - Model convergence issues due to complete separation (perfect prediction).

CCI - Charlson comorbidity index (modified); CDI - Clostridioides difficile infection; ELISA - Enzyme-linked immunosorbent assay; IgG - Immunoglobulin G; TNA - Toxin neutralization assay.

Supplementary Table 13. Variable importance of boosting model – ELISA and TNA measurements (Anti-toxin B IgG)

| Variable | Seroresponse - Toxin B (ELISA) | | | | Seroresponse - Toxin B (TNA) | | | |
| --- | --- | --- | --- | --- | --- | --- | --- | --- |
|  | Two-fold increase | Four-fold increase | Median fold increase | 75^th^ percentile fold increase | Two-fold increase | Four-fold increase | Median fold increase | 75^th^ percentile fold increase |
| Age  (>65 years) | 0.0022 | 0.0000 | 0.0000 | 0.0000 | 0.0000 | 0.0006 | 0.0028 | 0.0000 |
| Sex  (female) | 0.0000 | 0.0018 | 0.0030 | 0.0000 | 0.0051 | 0.0065 | 0.0056 | 0.0011 |
| Study region |  |  |  |  |  |  |  |  |
| North America | 0.0136 | 0.0134 | 0.0000 | 0.0049 | 0.0486 | 0.0413 | 0.0236 | 0.0074 |
| Western Europe | 0.0000 | 0.0000 | 0.0000 | 0.0005 | 0.0000 | 0.0000 | 0.0000 | 0.0000 |
| CCI (continuous) | 0.0091 | 0.0067 | 0.0041 | 0.0028 | 0.0005 | 0.0000 | 0.0005 | 0.0000 |
| CDI risk exposure  (future exposure) | 0.0013 | 0.0104 | 0.0000 | 0.0004 | 0.0191 | 0.0185 | 0.0000 | 0.0000 |
| Baseline IgG  (above median) | 0.0000 | 0.0000 | 0.0013 | 0.0000 | 0.0753 | 0.0917 | 0.1037 | 0.0637 |
| Vaccine group |  |  |  |  |  |  |  |  |
| 100 µg + Al(OH)_3_ | 0.1303 | 0.1051 | 0.0955 | 0.0326 | 0.0338 | 0.0284 | 0.0203 | 0.0227 |
| 100 µg | 0.0415 | 0.0421 | 0.0187 | 0.0050 | 0.0215 | 0.0185 | 0.0152 | 0.0093 |
| 50 µg + Al(OH)_3_ | 0.0383 | 0.0350 | 0.0178 | 0.0036 | 0.0137 | 0.0118 | 0.0089 | 0.0026 |
| 50 µg | 0.0309 | 0.0298 | 0.0197 | 0.0044 | 0.0065 | 0.0053 | 0.0032 | 0.0016 |

Variable importance is measured as in-bag risk reduction.

Al(OH)_3_ - Aluminium hydroxide; CCI - Charlson comorbidity index (modified); CDI - Clostridioides difficile infection; ELISA - Enzyme-linked immunosorbent assay;

IgG - Immunoglobulin G; TNA - Toxin neutralization assay.

Supplementary Table 14. Predictors of seroresponse against *C. difficile* Toxin A - Sensitivity analyses among participants with 0, 7, 30-day vaccination schedule

| Variable | Seroresponse - Toxin A (ELISA) | | | | Seroresponse - Toxin A (TNA) | | | |
| --- | --- | --- | --- | --- | --- | --- | --- | --- |
|  | Two-fold increase | Four-fold increase | Median  fold increase | 75^th^ percentile fold increase | Two-fold increase | Four-fold increase | Median  fold increase | 75^th^ percentile fold increase |
| Age  (>65 years) | - | - | LR*+MEM*  +BOOST | - | - | - | LR*+MEM* | CART |
| Sex  (female) | - | - | - | BOOST | - | LR+SLR+MEM  +BOOST | LR+SLR+MEM  +CART+BOOST | LR+SLR+MEM +BOOST |
| Study region  (versus others) |  |  |  |  |  |  |  |  |
| North America | - | - | - | - | - | - | - | BOOST |
| Western Europe | - | - | - | - | - | - | - | - |
| CCI (continuous) | - | - | LR*+MEM*+SLR  +CART+BOOST | LR*+MEM*  +BOOST | LR*+SLR+MEM* | LR+ SLR+MEM  +BOOST | LR +SLR+MEM  +CART+BOOST | CART+BOOST |
| CDI risk exposure  (future exposure) | LR+BOOST | LR+SLR  +BOOST | - | - | SLR+MEM  +BOOST | LR+SLR  +BOOST | - | BOOST |
| Baseline IgG  (above median) | - | - | - | - | - | LR+MEM | LR+SLR+MEM +CART+BOOST | LR+SLR+MEM  +CART+BOOST |
| Vaccine group  (versus placebo) |  |  |  |  |  |  |  |  |
| 100 µg + Al(OH)_3_ | LR+SLR+MEM  +CART+BOOST | LR+SLR+MEM +CART+BOOST | LR+SLR+MEM +CART+BOOST | LR+SLR+MEM +BOOST | CART+BOOST | CART+BOOST | CART+BOOST | CART+BOOST |
| 100 µg | LR+SLR+MEM +CART+BOOST | LR+SLR+MEM +CART+BOOST | LR+SLR+MEM +CART+BOOST | LR+SLR+MEM +BOOST | CART+BOOST | CART+BOOST | CART+BOOST | CART+BOOST |
| 50 µg + Al(OH)_3_ | LR+SLR+MEM +CART+BOOST | LR+SLR+MEM +CART+BOOST | LR+SLR+MEM +CART+BOOST | LR+SLR+MEM +BOOST | CART+BOOST | CART+BOOST | CART+BOOST | CART+BOOST |
| 50 µg | LR+SLR+MEM +CART+BOOST | LR+SLR+MEM +CART+BOOST | LR+SLR+MEM +CART+BOOST | LR+SLR+MEM +BOOST | CART+BOOST | CART+BOOST | CART+BOOST | CART+BOOST |

Al(OH)_3_ - Aluminium hydroxide; BOOST - Boosting model; CART - Classification and regression tree model; CCI - Charlson comorbidity index (modified); C. difficile - Clostridioides difficile; CDI - Clostridioides difficile infection; ELISA - Enzyme-linked immunosorbent assay (ELISA); IgG - Immunoglobulin G; LR - Logistic regression model; MEM - Mixed-effect model; SLR - Stepwise logistic regression model; TNA - Toxin neutralization assay.

The LR, SLR, and MEM model abbreviations are presented in the table if the respective variable is associated with a p-value <0.05 (grey color) or <0.01 (black color).

The CART model abbreviations are presented in the table if the respective variable is contributing with a mean impurity decrease >0.001 (grey color) or >10 (black color).

The BOOST model abbreviations are presented in the table if the respective variable is contributing with an in-bag risk reduction >0.001 (grey color) or >0.01 (black color).

*Due to collinearity, the presented association is conditional on selecting only Age or CCI in each of the LR or MEM multivariable models.

A hyphen (-) indicates that no association was found in any of the models.

Supplementary Table 15. Predictors of seroresponse against *C. difficile* Toxin B - Sensitivity analyses among participants with 0, 7, 30-day vaccination schedule

| Variable | Seroresponse - Toxin B (ELISA) | | | | Seroresponse - Toxin B (TNA) | | | |
| --- | --- | --- | --- | --- | --- | --- | --- | --- |
|  | Two-fold increase | Four-fold increase | Median  fold increase | 75^th^ percentile fold increase | Two-fold increase | Four-fold increase | Median  fold increase | 75^th^ percentile fold increase |
| Age  (>65 years) | LR*+SLR+MEM*  +BOOST | LR*+MEM* | - | - | LR*+SLR+MEM*  +BOOST | LR+SLR+MEM +CART+BOOST | LR*+SLR+MEM* +CART+BOOST | - |
| Sex  (female) | - | LR+SLR  +BOOST | LR+SLR+MEM  +BOOST | - | LR+SLR+MEM +CART+BOOST | LR+SLR+MEM +CART+BOOST | LR+ SLR+MEM  +BOOST | - |
| Study region  (versus others) |  |  |  |  |  |  |  |  |
| North America | BOOST | SLR+BOOST | - | LR+SLR+MEM  +BOOST | LR+SLR+MEM BOOST | LR+SLR+MEM +BOOST | LR+SLR+MEM  +CART+BOOST | BOOST |
| Western Europe | - | MEM | - | - | - | - | CART | - |
| CCI (continuous) | LR*+MEM* | LR+SLR+ MEM  +BOOST | LR+SLR+MEM  +CART+BOOST | BOOST | CART | CART | CART | - |
| CDI risk exposure  (future exposure) | - | BOOST | LR+SLR+MEM | - | LR+SLR  +BOOST | LR+SLR+MEM  +BOOST | CART | - |
| Baseline IgG  (above median) | LR+SLR+MEM | LR+SLR+MEM | LR+SLR+MEM  +BOOST | - | LR+SLR+MEM +CART+BOOST | LR+SLR+MEM +CART+BOOST | LR+SLR+MEM  +CART+BOOST | LR+SLR+MEM  +CART+BOOST |
| Vaccine group  (versus placebo) |  |  |  |  |  |  |  |  |
| 100 µg + Al(OH)_3_ | LR+SLR+MEM  +CART+BOOST | LR+SLR+MEM +CART+BOOST | LR+SLR+MEM +CART+BOOST | LR+SLR+MEM +BOOST | LR+SLR+MEM +CART+BOOST | LR+SLR+MEM +CART+BOOST | LR+SLR+MEM +CART+BOOST | LR+SLR+MEM +CART+BOOST |
| 100 µg | LR+SLR+MEM +CART+BOOST | LR+SLR+MEM +CART+BOOST | LR+SLR+MEM +CART+BOOST | LR+SLR+MEM +BOOST | LR+SLR+MEM +CART+BOOST | LR+SLR+MEM +CART+BOOST | LR+SLR+MEM +CART+BOOST | LR+SLR+MEM +CART+BOOST |
| 50 µg + Al(OH)_3_ | LR+SLR+MEM +CART+BOOST | LR+SLR+MEM +CART+BOOST | LR+SLR+MEM +CART+BOOST | LR+SLR+MEM +BOOST | LR+SLR+MEM +CART+BOOST | LR+SLR+MEM +CART+BOOST | LR+SLR+MEM +CART+BOOST | LR+SLR+MEM +CART+BOOST |
| 50 µg | LR+SLR+MEM +CART+BOOST | LR+SLR+MEM +CART+BOOST | LR+SLR+MEM +CART+BOOST | LR+SLR+MEM +BOOST | LR+SLR+MEM +CART+BOOST | LR+SLR+MEM +CART+BOOST | LR+SLR+MEM +CART+BOOST | LR+SLR+MEM +CART+BOOST |

Al(OH)_3_ - Aluminium hydroxide; BOOST - Boosting model; CART - Classification and regression tree model; CCI - Charlson comorbidity index (modified); C. difficile - Clostridioides difficile; CDI - Clostridioides difficile infection; ELISA - Enzyme-linked immunosorbent assay (ELISA); IgG - Immunoglobulin G; LR - Logistic regression model; MEM - Mixed-effect model; SLR - Stepwise logistic regression model; TNA - Toxin neutralization assay.

The LR, SLR, and MEM model abbreviations are presented in the table if the respective variable is associated with a p-value <0.05 (grey color) or <0.01 (black color).

The CART model abbreviations are presented in the table if the respective variable is contributing with a mean impurity decrease >0.001 (grey color) or >10 (black color).

The BOOST model abbreviations are presented in the table if the respective variable is contributing with an in-bag risk reduction >0.001 (grey color) or >0.01 (black color).

*Due to collinearity, the presented association is conditional on selecting only Age or CCI in each of the LR or MEM multivariable models.

A hyphen (-) indicates that no association was found in any of the models.

Supplementary Table 16. Charlson comorbidity index scoring system (Modified version)

| Condition | Weight | ICD-10 |
| --- | --- | --- |
| Myocardial infarction | 1 | I21.x – ST & NST-elevation myocardial infarction^*+^  I22.x – Subsequent ST & NST-elevation myocardial infarction^*+^  I25.2 – Old myocardial infarction^*+^ |
| Congestive heart failure | 1 | I09.9 – Rheumatic heart disease^*^  I11.0 – Hypertensive heart disease with heart failure^*+^  I13.0 – Hypertensive heart and chronic kidney disease with heart failure and stage 1 through stage 4 chronic kidney disease, or unspecified chronic kidney disease^*+^  I13.2 – Hypertensive heart and chronic kidney disease with heart failure and with stage 5 chronic kidney disease, or end stage renal disease^*+^  I25.5 – Ischemic cardiomyopathy^*+^  I42.0 – Cardiomyopathy^*+^  I42.5 – Other restrictive cardiomyopathy^*+^  I42.6 – Alcoholic cardiomyopathy^*+^  I42.7 – Cardiomyopathy due to drug and external agent^*+^  I42.8 – Other cardiomyopathies^*+^  I42.9 – Cardiomyopathy, unspecified^*+^  I43.x – Cardiomyopathy in diseases classified elsewhere^*+^  I50.x – Heart failure^*+^  P29.0 – Neonatal cardiac failure^*+^ |
| Peripheral vascular disease | 1 | I70.x – Atherosclerosis^*+^  I71.x – Aortic aneurysm and dissection^*+^  I73.1 – Thromboangiitis obliterans [Buerger's disease] ^*+^  I73.8 – Other specified peripheral vascular diseases^*+^  I73.9 – Peripheral vascular disease, unspecified^*+^  I77.1 – Stricture of artery^*+^  I79.0 – Aneurysm of aorta in diseases classified elsewhere^*+^  I79.1 – Aortitis in diseases classified elsewhere^+^  I79.2 – Peripheral angiopathy in diseases classified elsewhere^*+^  I79.8 – Other disorders of arteries, arterioles, and capillaries in diseases classified elsewhere^+^  K55.1 – Chronic vascular disorders of intestine^*+^  K55.8 – Other vascular disorders of intestine^*+^  K55.9 – Vascular disorder of intestine, unspecified^*+^  Z95.8 – Presence of other cardiac and vascular implants and grafts^*+^  Z95.9 – Presence of cardiac and vascular implant and graft, unspecified^*+^ |
| Cerebrovascular disease | 1 | G45.x – Transient cerebral ischemic attacks and related syndromes^*+^  G46.x – Vascular syndromes of brain in cerebrovascular diseases^*+^  H34.0x – Transient retinal artery occlusion^*+^  H34.1x – Central retinal artery occlusion^+^  H34.2x – Other retinal artery occlusions^+^  I60.x – Non-traumatic subarachnoid hemorrhage^*+^  I61.x – Non-traumatic intracerebral hemorrhage^*+^  I62.x – Other and unspecified non-traumatic intracranial hemorrhage^*+^  I63.x – Cerebral infarction^*+^  I64.x – Stroke, not specified as hemorrhage or infarction^*+^  I65.x – Occlusion and stenosis of precerebral arteries, not resulting in cerebral infarction^*+^  I66.x – Occlusion and stenosis of cerebral arteries, not resulting in cerebral infarction^*+^  I67.x – Other cerebrovascular diseases^*+^  I68.x – Cerebrovascular disorders in diseases classified elsewhere^*+^  I69.x – Sequelae of cerebrovascular disease^*^ |
| Dementia | 1 | F00.x – Dementia in Alzheimer disease^*^  F01.x – Vascular dementia^*+^  F02.x – Dementia in other diseases classified elsewhere^*+^  F03.x – Unspecified dementia^*+^  F04.x – Amnestic disorder due to known physiological condition^+^  F05.1 – Delirium superimposed on dementia^* /^ F05.x^+^  F06.1 – Catatonic disorder due to known physiological condition^+^  F06.8 – Other specified mental disorders due to known physiological condition^+^  G13.2 – Systemic atrophy primarily affecting the central nervous system in myxedema^+^  G13.8 – Systemic atrophy primarily affecting central nervous system in other diseases classified elsewhere^+^  G30.x – Alzheimer's disease^*+^  G31.1 – Senile degeneration of brain, not elsewhere classified^*+^  G31.2 – Degeneration of nervous system due to alcohol^+^  G91.4 – Hydrocephalus in diseases classified elsewhere^+^  G94 – Other disorders of brain in diseases classified elsewhere^+^  R41.81– Age-related cognitive decline^+^  R54 – Age-related physical debility^+^ |
| Chronic pulmonary disease | 1 | I27.8 – Other specified pulmonary heart diseases^*^  I27.9 – Pulmonary heart disease, unspecified^*^  J40.x – Bronchitis, not specified as acute or chronic^*+^  J41.x – Simple and mucopurulent chronic bronchitis^*+^  J42.x – Unspecified chronic bronchitis^*+^  J43.x – Emphysema^*+^  J44.x – Other chronic obstructive pulmonary disease^*+^  J45.x – Asthma^*+^  J46.x – Status asthmaticus^*+^  J47.x – Bronchiectasis^*+^  J60.x – Coal worker's pneumoconiosis^*+^  J61.x – Pneumoconiosis due to asbestos and other mineral fibers^*+^  J62.x – Pneumoconiosis due to dust containing silica^*+^  J63.x – Pneumoconiosis due to other inorganic dusts^*+^  J64.x – Unspecified pneumoconiosis^*+^  J65.x – Pneumoconiosis associated with tuberculosis^*+^  J66.x – Airway disease due to specific organic dust^*+^  J67.x – Hypersensitivity pneumonitis due to organic dust^*+^  J68.4 – Chronic respiratory conditions due to chemicals, gases, fumes and vapors^*+^  J70.1 – Chronic and other pulmonary manifestations due to radiation^*+^  J70.3 – Chronic drug-induced interstitial lung disorders^*+^ |
| Rheumatic disease | 1 | M05.x – Rheumatoid arthritis with rheumatoid factor^*+^  M06.x – Other rheumatoid arthritis^*+^  M31.5 – Giant cell arteritis with polymyalgia rheumatica^*+^  M32.x – Systemic lupus erythematosus (SLE) ^*+^  M33.x – Dermatopolymyositis^*+^  M34.x – Systemic sclerosis [scleroderma] ^*+^  M35.1 – Other overlap syndromes^*+^  M35.3 – Polymyalgia rheumatica^*+^  M36.0 – Dermato(poly)myositis in neoplastic disease^*+^ |
| Peptic ulcer disease | 1 | K25.x – Gastric ulcer^*+^  K26.x – Duodenal ulcer^*+^  K27.x – Peptic ulcer, site unspecified^*+^  K28.x – Gastrojejunal ulcer^*+^ |
| Diabetes without chronic complications | 1 | E08 – Diabetes mellitus due to underlying condition^+^  E09 – Drug or chemical induced diabetes mellitus^+^  E10.0-10.9 – Type 1 diabetes mellitus^*+^  E11.0-11.9 – Type 2 diabetes mellitus^*+^  E12.0-12.9 – Malnutrition-related diabetes mellitus^*^  E13.0-13.9 – Other specified diabetes mellitus^*+^  E14.0-14.9 – Unspecified diabetes mellitus^*^  (Subcategories: hyperosmolarity; ketoacidosis; other specified complications; unspecified complications; without complications)^+^ |
| Diabetes with chronic complications  Assumption: We could not distinguish diabetes sub-types and we used weights of 1 instead of 2 | 1 (2) | E08 – Diabetes mellitus due to underlying condition^+^  E09 – Drug or chemical induced diabetes mellitus^+^  E10.2-10.5, 10.7 – Type 1 diabetes mellitus^*^ / E10^+^  E11.2-11.5, 11.7 – Type 2 diabetes mellitus^*^ / E11^+^  E12.2-12.5, 12.7 – Malnutrition-related diabetes mellitus^*^ / E12^+^  E13.2-13.5, 13.7 – Other specified diabetes mellitus^*^ / E13^+^  E14.2-14.5, 14.7 – Unspecified diabetes mellitus^*^  (Subcategories: Diabetes mellitus due to underlying condition with kidney complications; Diabetes mellitus due to underlying condition with ophthalmic complications; Diabetes mellitus due to underlying condition with neurological complications; Diabetes mellitus due to underlying condition with circulatory complications)^+^ |
| Hemiplegia or paraplegia | 2 | G04.1 – Tropical spastic paraplegia^*+^  G11.4 – Hereditary spastic paraplegia^*+^  G80.0 – Spastic quadriplegic cerebral palsy^*+^  G80.1 – Spastic diplegic cerebral palsy^*+^  G80.2 – Spastic hemiplegic cerebral palsy^*+^  G81.x – Hemiplegia and hemiparesis^*+^  G82.x – Paraplegia (paraparesis) and quadriplegia (quadriparesis)^*+^  G83.0 – Diplegia of upper limbs^*^ / G83.x^+^  G83.1 – Monoplegia of lower limb^*^  G83.2 – Monoplegia of upper limb^*^  G83.3 – Monoplegia, unspecified^*^  G83.4 – Cauda equina syndrome^*^  G83.9 – Paralytic syndrome, unspecified^*^ |
| Any malignancy including lymphoma and leukemia, except malignant nonmelanoma neoplasm of skin | 2 | C0x.x – Malignant neoplasms of lip, oral cavity and pharynx^+^  C1x.x – Malignant neoplasms of lip, oral cavity and pharynx, esophagus, stomach, small intestine, colon^+^  C2x.x – Malignant neoplasms of rectum, anus, anal canal, liver and intrahepatic bile ducts, gallbladder, other and unspecified part of biliary tract, pancreas, other and ill-defined digestive organs^+^ / C00.x-C26.x^*^  C30.x – Malignant neoplasm of nasal cavity and middle ear^*+^  C31.x – Malignant neoplasm of accessory sinuses^*+^  C32.x – Malignant neoplasm of larynx^*+^  C33.x – Malignant neoplasm of trachea^*+^  C34.x – Malignant neoplasm of bronchus and lung^*+^  C37.x – Malignant neoplasm of thymus^*+^  C38.x – Malignant neoplasm of heart, mediastinum and pleura^*+^  C39.x – Malignant neoplasm of other and ill-defined sites in the respiratory system and intrathoracic organs^*+^  C40.x – Malignant neoplasm of bone and articular cartilage of limbs^*+^  C41.x – Malignant neoplasm of bone and articular cartilage of other and unspecified sites^*+^  C43.x – Malignant melanoma of skin^*+^  C45.x – Mesothelioma^*+^  C46.x – Kaposi's sarcoma^*+^  C47.x – Malignant neoplasm of peripheral nerves and autonomic nervous system^*+^  C48.x – Malignant neoplasm of retroperitoneum and peritoneum^*+^  C49.x – Malignant neoplasm of other connective and soft tissue  C50 – Malignant neoplasm of breast^*+^  C51-58.x – Malignant neoplasms of female genital organs^*+^  C60-63.x – Malignant neoplasm of male genital organs^*+^  C76.x – Malignant neoplasm of other and ill-defined sites^*+^  C81.x – Hodgkin lymphoma^*+^  C82.x – Follicular lymphoma^*+^  C83.x – Non-follicular lymphoma^*+^  C84.x – Mature T/NK-cell lymphomas^*+^  C85.x – Other specified and unspecified types of non-Hodgkin lymphoma^*+^  C88.x – Malignant immunoproliferative diseases and certain other B-cell lymphomas^*+^  C9x.x – Multiple myeloma and malignant plasma cell neoplasms, lymphoid leukemia, monocytic leukemia, other leukemias of specified cell type, leukemia of unspecified cell type, other and unspecified malignant neoplasms of lymphoid, hematopoietic and related tissue^+^ / C90.x-97.x^*^ |
| Metastatic solid tumor  Assumption: We could not distinguish metastatic solid tumor and we used weights of 2 instead of 6. Likely, severely ill patients would confine with the trial inclusion criteria. | 2 (6) | C77.x – Secondary and unspecified malignant neoplasm of lymph nodes^*+^  C78.x – Secondary malignant neoplasm of respiratory and digestive organs^*+^  C79.x – Secondary malignant neoplasm of other and unspecified sites^*+^  C80.x – Malignant neoplasm^*+^ |
| Liver disease (mild) | 1 | B18.x – Chronic viral hepatitis^*+^  K70.0 – Alcoholic fatty liver^*+^  K70.1 – Alcoholic hepatitis^*+^  K70.2 – Alcoholic fibrosis and sclerosis of liver^*+^  K70.3 – Alcoholic cirrhosis of liver^*+^  K70.9 – Alcoholic liver disease, unspecified^*+^  K71.3 – Toxic liver disease with chronic persistent hepatitis^*+^  K71.4 – Toxic liver disease with chronic lobular hepatitis^*+^  K71.5 – Toxic liver disease with chronic active hepatitis^*+^  K71.7 – Toxic liver disease with fibrosis and cirrhosis of liver^*+^  K73.x – Chronic hepatitis, not elsewhere classified^*+^  K74.x – Fibrosis and cirrhosis of liver^*+^  K76.0 – Fatty (change of) liver, not elsewhere classified^*+^  K76.2 – Central hemorrhagic necrosis of liver^*+^  K76.3 – Infarction of liver^*+^  K76.4 – Peliosis hepatis^*+^  K76.8 – Other specified diseases of liver^*+^  K76.9 – Liver disease, unspecified^*+^  Z94.4 – Liver transplant status^*+^ |
| Liver disease (moderate-severe) | 3 | I85.0x – Esophageal varices^*+^  I85.9 – Oesophageal varices without bleeding^*^  I86.4 – Gastric varices^*+^  I98.2 – Oesophageal varices without bleeding in diseases classified elsewhere^*^  K70.4x – Alcoholic hepatic failure^*+^  K71.1x – Toxic liver disease with hepatic necrosis^*+^  K72.1x – Chronic hepatic failure^*+^  K72.9x – Hepatic failure, unspecified^*+^  K76.5 – Hepatic veno-occlusive disease^*+^  K76.6 – Portal hypertension^*+^  K76.7 – Hepatorenal syndrome^*+^ |
| Renal disease (mild-moderate)  Assumption: We could not distinguish renal disease stages and we used weights of 2 instead of 1. | 2 (1) | I12.0 – Hypertensive chronic kidney disease with stage 5 chronic kidney disease or end stage renal disease^*^  I12.9 – Hypertensive chronic kidney disease with stage 1 through stage 4 chronic kidney disease, or unspecified chronic kidney disease^+^  I13.0 – Hypertensive heart and chronic kidney disease with heart failure and stage 1 through stage 4 chronic kidney disease, or unspecified chronic kidney disease^+^  I13.1 – Hypertensive heart and CKD without heart failure with stage 1 through stage 4 chronic kidney disease, or unspecified chronic kidney disease^*+^  N03.3-3.7 – Chronic nephritic syndrome^*^ / N03.x^+^  N05.2-2.7 – Unspecified nephritic syndrome^*^ / N05.x^+^  N18.1 – Chronic kidney disease, Stage^+^ / N18.x^*^  N18.2 – Chronic kidney disease, Stage 2 (mild)^+^  N18.3 – Chronic kidney disease, Stage 3 (moderate)^+^  N18.4 – Chronic kidney disease, Stage 4 (severe)^+^  N18.9 – Chronic kidney disease, unspecifie^+^  N19.x – Unspecified kidney failure^*^  N25.0 – Renal osteodystrophy^*^  Z49.0-Z49.2 – Preparatory care for renal dialysis/Encounter for fitting and adjustment of extracorporeal/peritoneal dialysis catheter^*^  Z94.0 – Kidney transplant status^*+^  Z99.2 – Dependence on renal dialysis^*^ |
| Renal disease (severe)  Assumption: We could not distinguish renal disease stages and we used weights of 2 instead of 3. | 2 (3) | I12.0 – Hypertensive chronic kidney disease with stage 5 chronic kidney disease or end stage renal disease^*+^  I13.1 – Hypertensive heart and CKD without heart failure with stage 5 chronic kidney disease, or end stage renal disease^*+^  I13.2 – Hypertensive heart and chronic kidney disease with heart failure and with stage 5 chronic kidney disease, or end stage renal disease^+^  N03.2-N03.7/ N05.2-N05.7 – Chronic nephritic syndrome types^*^  N18.5 – Chronic kidney disease, Stage 5 / N18.x^*+^  N18.6 – End stage renal disease^+^  N19.x – Unspecified kidney failure^*+^  N25.0 – Renal osteodystrophy^*+^  Z49.x – Encounter for care involving renal dialysis / Z49.0-Z49.2^*+^  Z99.2 – Dependence on renal dialysis^*+^ |
| HIV infection, no AIDS  *No observations detected.* | 3 | B20.x-22.x, B24.x – Human immunodeficiency virus [HIV] disease^*^ / B20.x^+^ |
| AIDS (HIV Infection + opportunistic infection)  *No observations detected.* | 6 | A02.1 – Salmonella septicemia, recurrent^+^  A07.2 – Cryptosporidiosis, chronic intestinal (greater than 1 month's duration)^+^  A07.3 – Isosporiasis, chronic intestinal (greater than 1 month's duration)^+^  A15-A19 – Tuberculosis^+^  A31.x – Mycobacterium avium comp^+^  A81.2 – Progressive multifocal leukoencephalopathy^+^  B00 – Herpes simplex: chronic ulcer(s) (greater than 1 month's duration); or bronchitis, pneumonitis, or esophagitis^+^  B25.x – Cytomegalovirus disease (particularly CMV retinitis) ^+^  B37.x – Candidiasis of bronchi, trachea, esophagus, or lungs^+^  B38.x – Coccidioidomycosis^+^  B39.x – Histoplasmosis G93.4x – Encephalopathy, HIV-related^+^  B45.x – Cryptococcosis^+^  B58.x – Toxoplasmosis of brain^+^  B59 – Pneumocystis carinii pneumonia^+^  C53.x – Invasive cervical cancer^+^  C46.x – Kaposi's sarcoma^+^  C81-C96 – Lymphoma, multiple forms^+^  G93.4x – Encephalopathy, HIV-related^+^  R64 – Wasting syndrome due to HIV^+^  Z87.01 – Pneumonia, recurrent^+^ |

Source:

*Quan H, Sundararajan V, Halfon P, et al. Coding algorithms for defining Comorbidities in ICD-9-CM and ICD-10 administrative data. Med Care. 2005 Nov; 43(11): 1130-9.

^+^Glasheen WP, Cordier T, Gumpina R, Haugh G, Davis J, Renda A. Charlson Comorbidity Index: ICD-9 Update and ICD-10 Translation. Am Health Drug Benefits. 2019 Jun-Jul;12(4):188-97.

Supplementary Table 17. Frequency of the comorbidities and age groups used in deriving the modified Charlson comorbidity index

| Conditions | Frequency n (%)  N = 1,096 |
| --- | --- |
| Myocardial infarction | 1 (0.1) |
| Congestive heart failure | 98 (8.9) |
| Peripheral vascular disease | 20 (1.8) |
| Cerebrovascular disease | 16 (1.5) |
| Dementia | 2 (0.2) |
| Chronic pulmonary disease | 179 (16.3) |
| Connective tissue disease | 10 (0.9) |
| Peptic ulcer disease | 18 (1.6) |
| Liver disease (mild/moderate-severe) | 18 (1.6) |
| Diabetes mellitus (w/wo chronic complications) | 302 (27.6) |
| Hemiplegia or paraplegia | 2 (0.2) |
| Renal disease (mild-moderate/severe) | 72 (6.6) |
| Malignancy/Solid tumor | 9 (0.8) |
| HIV/AIDS | 0 (0.0) |
| Age >65 years | 505 (46.1) |

AIDS- Acquired immunodeficiency syndrome; HIV - Human immunodeficiency virus; w/wo – With or without.

References

1. Quan, H. *et al.* Coding algorithms for defining comorbidities in ICD-9-CM and ICD-10 administrative data. *Medical care* **43,** 1130–1139; 10.1097/01.mlr.0000182534.19832.83 (2005).

2. Glasheen, W. P. *et al.* Charlson Comorbidity Index: ICD-9 Update and ICD-10 Translation. *American Health & Drug Benefits* **12,** 188–197 (2019).

3. R Core Team (2024). R: A Language and Environment for Statistical Computing_. R Foundation for Statistical Computing, Vienna, Austria. (Available online: www.R-project.org)

4. Venables, W. N. & Ripley, B. D. *Modern applied statistics with S* (Springer, New York, NY, 2002).

5. Bates, D., Mächler, M., Bolker, B. & Walker, S. Fitting Linear Mixed-Effects Models Using lme4. *J. Stat. Soft.* **67**; 10.18637/jss.v067.i01 (2015).

6. Therneau, T. Atkinson, B. and Ripley, B. (2015). rpart: Recursive Partitioning and Regression Trees. R package version 4.1.23. (Available online: CRAN.R-project.org/package=rpart)

7. Hofner, B., Mayr, A., Robinzonov, N. & Schmid, M. Model-based boosting in R: a hands-on tutorial using the R package mboost. *Comput Stat* **29,** 3–35; 10.1007/s00180-012-0382-5 (2014).

8. Robin, X. *et al.* pROC: an open-source package for R and S+ to analyze and compare ROC curves. *BMC bioinformatics* **12,** 77; 10.1186/1471-2105-12-77 (2011).

9. Kuhn, M. Building Predictive Models in R Using the caret Package. *J. Stat. Soft.* **28**; 10.18637/jss.v028.i05 (2008).

10. Lüdecke, D. (2024). sjPlot: Data Visualization for Statistics in Social Science. R package version 2.8.17. (Available online: www.CRAN.R-project.org/package=sjPlot)

11. Wozniak, E. (2015). Table1: Summary Table. R package version 1.0. (Available online: www.github.com/emwozniak/Table1)
